# Supplementary material for: Participation and returns from informal service-oriented non-farm enterprises: Evidence from a survey of Nigerian households
Source: PLoS One. 2024 Mar 18;19(3):e0298794. doi: 10.1371/journal.pone.0298794 (PMC10947675; doi:10.1371/journal.pone.0298794)
Supplement: S1 Appendix — (DOCX) [file pone.0298794.s001.docx]

**Appendix**

Appendix A1: Description of Informal Non-Farm Household Enterprises

| **Wholesale, Retail and Trade/ Repairs** |
| --- |
| Wholesale and retail trade |
| Repairs of vehicles |
| **Personal and Consultancy** |
| Transportation services |
| Warehousing and support activities |
| Accommodation |
| Publishing activities |
| Motion picture, video, broadcasting and television programmes |
| Telecommunications, computer programming. |
| Financial, real estate and legal services activities. |
| Scientific research, advertising research a |
| Veterinary activities |
| Rental and leasing, travel agency, reservation |
| Education |
| Human health and social works activities |
| Residential care activities |
| Other personal service activities (barbers, launders etc) |
| Beauticians and tailoring |

Source: Culled from the Nigerian GHS-Panel Data (NBS 2010 – 2015)

Table A2: Determinants of Wholesale and Retail Oriented Household Enterprises Home and Non-Home based (Pooled and RE-Probit Models)

|  | HBE | | | | |  | | Non-HBE | | | | | |  |
| --- | --- | --- | --- | --- | --- | --- | --- | --- | --- | --- | --- | --- | --- | --- |
|  | Pooled-Probit | |  | RE-Probit | | |  | | Pooled-Probit | |  | RE-Probit | | |
| Variables | Coefficient  1 | AME  2 |  | Coefficient  3 | AME  4 | |  | | Coefficient  5 | AME  6 |  | Coefficient  7 | AME  8 | |
| Age Group (Base <=25) |  |  |  |  |  | |  | |  |  |  |  |  | |
| 26 - 35 | 0.223** | 0.080** |  | 0.665* | 0.076* | |  | | 0.269*** | 0.102*** |  | 1.141*** | 0.126*** | |
|  | (0.097) | (0.035) |  | (0.344) | (0.041) | |  | | (0.095) | (0.037) |  | (0.372) | (0.034) | |
| 36 - 45 | 0.298*** | 0.105*** |  | 0.853** | 0.092** | |  | | 0.516*** | 0.185*** |  | 1.949*** | 0.216*** | |
|  | (0.099) | (0.036) |  | (0.369) | (0.043) | |  | | (0.094) | (0.035) |  | (0.392) | (0.031) | |
| 46 - 55 | 0.251** | 0.089** |  | 0.893** | 0.095** | |  | | 0.503*** | 0.181*** |  | 2.070*** | 0.226*** | |
|  | (0.111) | (0.040) |  | (0.421) | (0.046) | |  | | (0.101) | (0.037) |  | (0.427) | (0.033) | |
| > 55 | 0.556*** | 0.181*** |  | 1.979*** | 0.141*** | |  | | 0.609*** | 0.213*** |  | 2.400*** | 0.247*** | |
|  | (0.113) | (0.037) |  | (0.467) | (0.043) | |  | | (0.107) | (0.038) |  | (0.458) | (0.031) | |
| Education (Base = Others) |  |  |  |  |  | |  | |  |  |  |  |  | |
| Primary | 0.221** | 0.076** |  | 0.488 | 0.046 | |  | | 0.068 | 0.023 |  | 0.529 | 0.061 | |
|  | (0.097) | (0.035) |  | (0.354) | (0.037) | |  | | (0.113) | (0.038) |  | (0.404) | (0.050) | |
| Secondary | 0.232** | 0.080** |  | 0.578 | 0.053 | |  | | -0.032 | -0.011 |  | 0.211 | 0.026 | |
|  | (0.102) | (0.036) |  | (0.387) | (0.039) | |  | | (0.115) | (0.040) |  | (0.417) | (0.052) | |
| Post-Secondary | 0.410*** | 0.134*** |  | 1.002** | 0.076* | |  | | 0.347** | 0.106** |  | 1.335** | 0.119** | |
|  | (0.134) | (0.043) |  | (0.511) | (0.040) | |  | | (0.136) | (0.043) |  | (0.537) | (0.052) | |
| Male_owner | -0.762*** | -0.267*** |  | -3.308*** | -0.285*** | |  | | -1.150*** | -0.367*** |  | -5.058*** | -0.347*** | |
|  | (0.065) | (0.023) |  | (0.408) | (0.021) | |  | | (0.057) | (0.017) |  | (0.415) | (0.016) | |
| Zones (Base= North-Cent) |  |  |  |  |  | |  | |  |  |  |  |  | |
| North-East | 0.126 | 0.043 |  | 0.040 | 0.003 | |  | | 0.365*** | 0.099*** |  | 2.409*** | 0.102** | |
|  | (0.118) | (0.041) |  | (0.490) | (0.042) | |  | | (0.127) | (0.032) |  | (0.661) | (0.045) | |
| North-West | 0.174 | 0.059 |  | 0.110 | 0.009 | |  | | 0.394*** | 0.106*** |  | 2.390*** | 0.102** | |
|  | (0.110) | (0.038) |  | (0.468) | (0.039) | |  | | (0.111) | (0.028) |  | (0.620) | (0.044) | |
| South-East | 0.166 | 0.057 |  | 0.402 | 0.028 | |  | | -0.062 | -0.020 |  | -0.199 | -0.021 | |
|  | (0.134) | (0.045) |  | (0.548) | (0.038) | |  | | (0.091) | (0.029) |  | (0.476) | (0.050) | |
| South-South | 0.379*** | 0.121*** |  | 0.895** | 0.049 | |  | | -0.015 | -0.005 |  | -0.027 | -0.003 | |
|  | (0.110) | (0.036) |  | (0.451) | (0.031) | |  | | (0.091) | (0.029) |  | (0.470) | (0.047) | |
| South-West | -0.008 | -0.003 |  | -0.534 | -0.056 | |  | | -0.516*** | -0.186*** |  | -1.790*** | -0.201*** | |
|  | (0.109) | (0.039) |  | (0.452) | (0.047) | |  | | (0.089) | (0.031) |  | (0.445) | (0.041) | |
| Rural | -0.038 | -0.012 |  | -0.169 | -0.013 | |  | | -0.045 | -0.015 |  | 0.142 | 0.015 | |
|  | (0.075) | (0.024) |  | (0.305) | (0.022) | |  | | (0.061) | (0.020) |  | (0.285) | (0.031) | |
| HH-size | 0.014* | 0.005* |  | 0.034 | 0.003 | |  | | 0.016* | 0.005* |  | 0.059 | 0.006 | |
|  | (0.009) | (0.003) |  | (0.036) | (0.003) | |  | | (0.009) | (0.003) |  | (0.037) | (0.004) | |
| Own_home | 0.058 | 0.019 |  | 0.060 | 0.005 | |  | | -0.062 | -0.020 |  | -0.292 | -0.031 | |
|  | (0.073) | (0.024) |  | (0.273) | (0.022) | |  | | (0.060) | (0.020) |  | (0.233) | (0.024) | |
| Electric | -0.149** | -0.048** |  | -0.625** | -0.044** | |  | | -0.096 | -0.032 |  | -0.261 | -0.027 | |
|  | (0.070) | (0.023) |  | (0.268) | (0.019) | |  | | (0.063) | (0.020) |  | (0.227) | (0.023) | |
| Transport | -0.010 | -0.003 |  | -0.110 | -0.009 | |  | | 0.020 | 0.007 |  | 0.049 | 0.005 | |
|  | (0.084) | (0.028) |  | (0.258) | (0.021) | |  | | (0.071) | (0.024) |  | (0.224) | (0.024) | |
| Micro-finance | 0.061 | 0.020 |  | -0.356 | -0.031 | |  | | -0.042 | -0.014 |  | 0.280 | 0.028 | |
|  | (0.102) | (0.033) |  | (0.300) | (0.030) | |  | | (0.091) | (0.031) |  | (0.278) | (0.026) | |
| Profit | -0.000 | -0.000 |  | -0.000 | -0.000 | |  | | 0.000 | 0.000 |  | 0.000 | 0.000 | |
|  | (0.000) | (0.000) |  | (0.000) | (0.000) | |  | | (0.000) | (0.000) |  | (0.000) | (0.000) | |
| Paid_hh | 0.073 | 0.024 |  | 0.320 | 0.023* | |  | | 0.053 | 0.018 |  | 0.021 | 0.002 | |
|  | (0.066) | (0.021) |  | (0.199) | (0.014) | |  | | (0.056) | (0.018) |  | (0.164) | (0.018) | |
| Unpaid_hh | 0.168*** | 0.054*** |  | 0.334* | 0.024* | |  | | 0.089 | 0.029 |  | 0.288* | 0.030* | |
|  | (0.063) | (0.020) |  | (0.194) | (0.014) | |  | | (0.057) | (0.019) |  | (0.166) | (0.017) | |
| Constant | 0.101 |  |  | 2.699*** |  | |  | | 0.789*** |  |  | 2.972*** |  | |
|  | (0.176) |  |  | (0.703) |  | |  | | (0.178) |  |  | (0.707) |  | |
| Log Likelihood | -1257 |  |  | -1085.33 |  | |  | | -1632.0 |  |  | -1360 |  | |
| $\hat{\rho}$(rho) |  |  |  | 0.960 |  | |  | |  |  |  | 0.952 |  | |
|  |  |  |  | (0.004) |  | |  | |  |  |  | (0.100) |  | |
| Likelihood ratio |  |  |  | 343.39 |  | |  | |  |  |  | 545.0 |  | |
| P-value |  |  |  | 0.000 |  | |  | |  |  |  | 0.000 |  | |
| Observations | 2,278 | 2,278 |  | 2,278 | 2,278 | |  | | 3,116 | 3,116 |  | 3,116 | 3,116 | |
| Number of id |  |  |  | 1,691 |  | |  | |  |  |  | 2,112 |  | |

Robust standard errors in parentheses; ***, ** and * significant at 1%, 5% and 10% respectively.

Note: AME=Average Marginal Effects.

Source: Authors’ Computation using GHS Cross-Sectional Panel Data (2010 – 2015).

Table A3: Determinants of Productivity Estimates for Informal HBEs using (Pooled OLS, Between Effects and Random Effects Models)

|  | **Pooled OLS MODEL** | | | **RANDOM EFFECT MODEL** | | |
| --- | --- | --- | --- | --- | --- | --- |
| Variables | Wholesale and Retail Trade  1 | Consultancy & Personal Services  2 | Both  3 | Wholesale and Retail Trade  7 | Consultancy & Personal Services  8 | Both  9 |
|  |  |  |  |  |  |  |
| WRT (C&P =0) |  |  | -0.358*** |  |  | -0.349*** |
|  |  |  | (0.065) |  |  | (0.069) |
| Age Group (Base <=25) |  |  |  |  |  |  |
| 26 – 35 | 0.072 | 0.579*** | 0.094 | 0.119 | 0.208 | 0.128 |
|  | (0.120) | (0.189) | (0.106) | (0.116) | (0.167) | (0.095) |
| 36 – 45 | 0.133 | 1.222*** | 0.263** | 0.188 | 0.442** | 0.253*** |
|  | (0.118) | (0.251) | (0.102) | (0.120) | (0.181) | (0.098) |
| 46 – 55 | 0.162 | 0.209 | 0.070 | 0.309** | 0.103 | 0.242** |
|  | (0.144) | (0.214) | (0.163) | (0.135) | (0.203) | (0.109) |
| > 55 | -0.069 | 1.466*** | 0.051 | 0.127 | 0.243 | 0.160 |
|  | (0.160) | (0.359) | (0.150) | (0.134) | (0.223) | (0.107) |
| Education (Base = Others) |  |  |  |  |  |  |
| Primary | 0.271*** | 0.320** | 0.285*** | 0.032 | 0.041 | 0.013 |
|  | (0.081) | (0.126) | (0.070) | (0.115) | (0.174) | (0.092) |
| Secondary | 0.472*** | 0.850*** | 0.529*** | 0.332*** | 0.433** | 0.326*** |
|  | (0.136) | (0.213) | (0.138) | (0.125) | (0.190) | (0.101) |
| Post-Secondary | 0.007 | -0.456** | -0.056 | 0.603*** | 1.006*** | 0.665*** |
|  | (0.110) | (0.192) | (0.089) | (0.160) | (0.252) | (0.137) |
| Male_owner | 1.065*** | -0.562* | 0.895*** | 0.734*** | 0.587*** | 0.691*** |
|  | (0.181) | (0.305) | (0.173) | (0.089) | (0.130) | (0.074) |
| Zones (Base= North-Cent) |  |  |  |  |  |  |
| North-East | -0.738*** | 0.136 | -0.520** | -0.898*** | -0.882*** | -0.881*** |
|  | (0.168) | (0.328) | (0.219) | (0.147) | (0.255) | (0.134) |
| North-West | -0.231 | 0.564 | -0.072 | -0.483*** | -0.730*** | -0.566*** |
|  | (0.165) | (0.352) | (0.227) | (0.139) | (0.224) | (0.118) |
| South-East | -0.035 | 0.161 | -0.074 | 0.024 | -0.104 | -0.015 |
|  | (0.159) | (0.305) | (0.154) | (0.166) | (0.298) | (0.144) |
| South-South | 0.064 | -0.684** | -0.197 | 0.419*** | 0.129 | 0.353*** |
|  | (0.205) | (0.291) | (0.311) | (0.132) | (0.231) | (0.114) |
| South-West | -0.409** | -2.431*** | -0.790** | -0.114 | -0.313 | -0.168 |
|  | (0.196) | (0.578) | (0.330) | (0.140) | (0.216) | (0.114) |
| Rural | -0.098 | -1.005*** | -0.241* | 0.035 | -0.078 | 0.003 |
|  | (0.105) | (0.276) | (0.137) | (0.090) | (0.155) | (0.077) |
| HH-size | -0.043*** | 0.006 | -0.040*** | -0.034*** | -0.022 | -0.031*** |
|  | (0.011) | (0.021) | (0.009) | (0.010) | (0.018) | (0.010) |
| Electric | 0.238*** | -0.327* | 0.151** | 0.229*** | -0.033 | 0.165** |
|  | (0.079) | (0.174) | (0.069) | (0.082) | (0.141) | (0.073) |
| Transport | 0.035 | 0.245* | 0.057 | 0.044 | 0.163 | 0.064 |
|  | (0.089) | (0.138) | (0.076) | (0.094) | (0.150) | (0.075) |
| Micro-finance | 0.148 | 0.925*** | 0.301** | 0.078 | 0.578*** | 0.188* |
|  | (0.123) | (0.255) | (0.137) | (0.109) | (0.182) | (0.110) |
| Turnover | 0.050*** | 0.098*** | 0.060*** | 0.052*** | 0.113*** | 0.061*** |
|  | (0.013) | (0.035) | (0.013) | (0.011) | (0.027) | (0.013) |
| Paid_hh | -0.504*** | -0.427*** | -0.511*** | -0.357*** | -0.455*** | -0.377*** |
|  | (0.080) | (0.120) | (0.065) | (0.070) | (0.116) | (0.061) |
| Inverse mills ratio | **S** | **S** | **S** | **NS** | **NS** | **NS** |
| (lambda) |  |  |  |  |  |  |
| Constant | 9.640*** | 14.583*** | 10.279*** | 8.532*** | 8.308*** | 8.598*** |
|  | (0.504) | (1.564) | (0.852) | (0.213) | (0.324) | (0.171) |
|  |  |  |  |  |  |  |
| Observations | 1,547 | 526 | 2,073 | 1,547 | 526 | 2,073 |
| R-squared | 0.258 | 0.375 | 0.259 | 0.254 | 0.290 | 0.260 |
| Number of id |  |  |  | 1,160 | 427 | 1,553 |
| LM Test Statistic |  |  |  | 46.810 | 16.92 | 62.31 |
| P-values |  |  |  | 0.000 | 0.000 | 0.000 |

Robust standard errors in parentheses; ***, ** and * significant at 1%, 5% and 10% respectively. ; C&P=Consultancy and Personal Services; S implies that the selectivity term is significant in applied model, otherwise, NS (insignificant).

Source: Authors’ Computation using GHS Cross-Sectional Panel Data (2010 – 2015).

Table A4: Determinants of Productivity Estimates for Informal non-HBEs using (Pooled OLS and Random Effects Models)

|  | **Pooled OLS MODEL** | | | **RANDOM EFFECT MODEL** | | |
| --- | --- | --- | --- | --- | --- | --- |
| Variables | Wholesale and Retail Trade  1 | Consultancy & Personal Services  2 | Both  3 | Wholesale and Retail Trade  7 | Consultancy & Personal Services  8 | Both  9 |
|  |  |  |  |  |  |  |
| C&P (WRT=0) |  |  | -0.432*** |  |  | -0.394*** |
|  |  |  | (0.059) |  |  | (0.064) |
| Age Group (Base <=25) |  |  |  |  |  |  |
| 26 – 35 | 0.227** | 0.660*** | 0.146* | 0.209* | 0.027 | 0.143* |
|  | (0.107) | (0.169) | (0.087) | (0.120) | (0.153) | (0.086) |
| 36 – 45 | 0.449*** | 1.286*** | 0.323*** | 0.406*** | 0.058 | 0.298*** |
|  | (0.105) | (0.227) | (0.087) | (0.120) | (0.160) | (0.089) |
| 46 – 55 | 0.444*** | 0.228 | 0.375*** | 0.423*** | 0.235 | 0.344*** |
|  | (0.118) | (0.173) | (0.098) | (0.129) | (0.175) | (0.102) |
| > 55 | 0.451*** | 1.070*** | 0.308*** | 0.479*** | 0.034 | 0.342*** |
|  | (0.122) | (0.226) | (0.102) | (0.134) | (0.185) | (0.105) |
| Education (Base = Others) |  |  |  |  |  |  |
| Primary | -0.043 | -0.913*** | 0.029 | -0.001 | 0.214 | 0.050 |
|  | (0.132) | (0.182) | (0.113) | (0.133) | (0.225) | (0.120) |
| Secondary | 0.237* | -1.465*** | 0.254** | 0.230* | 0.288 | 0.237* |
|  | (0.139) | (0.328) | (0.119) | (0.137) | (0.225) | (0.124) |
| Post-Secondary | 0.643*** | -3.862*** | 0.629*** | 0.642*** | 0.566** | 0.616*** |
|  | (0.157) | (0.577) | (0.135) | (0.159) | (0.266) | (0.142) |
| Male_owner | 0.467*** | 1.341*** | 0.504*** | 0.453*** | 0.559*** | 0.488*** |
|  | (0.071) | (0.153) | (0.058) | (0.077) | (0.114) | (0.064) |
| Zones (Base= North-Cent) |  |  |  |  |  |  |
| North-East | -0.471*** | 3.640*** | -0.411*** | -0.464*** | -0.218 | -0.423*** |
|  | (0.161) | (0.609) | (0.133) | (0.157) | (0.249) | (0.141) |
| North-West | -0.089 | 1.052*** | -0.168 | -0.051 | -0.360 | -0.141 |
|  | (0.146) | (0.312) | (0.121) | (0.143) | (0.223) | (0.128) |
| South-East | -0.400*** | -1.797*** | -0.298*** | -0.403*** | 0.018 | -0.291*** |
|  | (0.102) | (0.321) | (0.085) | (0.112) | (0.177) | (0.090) |
| South-South | 0.069 | -0.828*** | 0.146* | 0.085 | 0.376** | 0.164* |
|  | (0.104) | (0.241) | (0.087) | (0.112) | (0.177) | (0.092) |
| South-West | -0.260** | -5.174*** | -0.245*** | -0.245** | -0.201 | -0.232** |
|  | (0.109) | (0.766) | (0.088) | (0.113) | (0.164) | (0.094) |
| Rural | -0.161** | -2.014*** | -0.135** | -0.160** | -0.172 | -0.144** |
|  | (0.069) | (0.312) | (0.060) | (0.075) | (0.123) | (0.066) |
| HH-size | 0.000 | -0.090*** | -0.000 | 0.001 | -0.010 | -0.002 |
|  | (0.010) | (0.020) | (0.008) | (0.011) | (0.017) | (0.009) |
| Electric | 0.192*** | 0.342*** | 0.189*** | 0.171** | 0.084 | 0.155** |
|  | (0.072) | (0.114) | (0.061) | (0.072) | (0.112) | (0.061) |
| Transport | 0.060 | 0.056 | 0.061 | 0.084 | 0.081 | 0.081 |
|  | (0.078) | (0.108) | (0.064) | (0.077) | (0.113) | (0.061) |
| Micro-finance | -0.063 | 0.340** | -0.010 | -0.140 | 0.121 | -0.064 |
|  | (0.110) | (0.133) | (0.087) | (0.103) | (0.139) | (0.085) |
| Turnover | 0.004*** | 0.004 | 0.004*** | 0.004*** | 0.004 | 0.004*** |
|  | (0.000) | (0.003) | (0.000) | (0.001) | (0.005) | (0.000) |
| Paid_hh | -0.457*** | -0.246** | -0.484*** | -0.318*** | -0.410*** | -0.352*** |
|  | (0.065) | (0.105) | (0.053) | (0.060) | (0.086) | (0.050) |
| Inverse mills ratio | **NS** | **S** | **NS** | **NS** | **NS** | **NS** |
| (lambda) |  |  |  |  |  |  |
| Constant | 9.023*** | 20.371*** | 9.027*** | 8.984*** | 8.681*** | 9.024*** |
|  | (0.199) | (1.759) | (0.170) | (0.210) | (0.346) | (0.179) |
|  |  |  |  |  |  |  |
| Observations | 2,012 | 778 | 2,790 | 2,012 | 778 | 2,790 |
| R-squared | 0.134 | 0.198 | 0.136 | 0.132 | 0.136 | 0.133 |
| Number of id |  |  |  | 1,406 | 585 | 1,937 |
| LM-Test statistic |  |  |  | 71.89 | 21.08 | 104.24 |
| P-values |  |  |  | 0.000 | 0.000 | 0.000 |

Robust standard errors in parentheses; ***, ** and * significant at 1%, 5% and 10% respectively; WRT=Wholesale, Retail and Trade; C&P=Consultancy and Personal Services; S implies that the selectivity term is significant in applied model, otherwise, NS (insignificant).

Source: Authors’ Computation using GHS Cross-Sectional Panel Data (2010 – 2015).

Table A5: Coefficients of the selectivity terms from all models

|  | Pooled OLS | | | | | |  | RANDOM EFFECTS MODEL | | | | | |  |
| --- | --- | --- | --- | --- | --- | --- | --- | --- | --- | --- | --- | --- | --- | --- |
|  | Wholesale and Retail Trade | | Consultancy & Personal Services | | Both | |  | Wholesale and Retail Trade | | Consultancy & Personal Services | | Both | |  |
|  |  | |  | |  | |  |  | |  | |  | |  |
| HBE and non-HBE (Combined) | |  | |  | |  | |  |  | |  | |  | |
| Inverse mills ratio  (lambda) | -0.072 | | -3.007 | | -0.952 | |  | 1.250*** | | 0.167 | | 0.894** | |  |
|  | (0.565) | | (2.064) | | (0.883) | |  | (0.462) | | (0.666) | | (0.382) | |  |
| HBE |  | |  | |  | |  |  | |  | |  | |  |
| Inverse mills ratio(lambda) | -1.461** | | -6.180*** | | -3.091** | |  | 0.236 | | -0.184 | | -0.267 | |  |
|  | (0.632) | | (1.547) | | (1.478) | |  | (0.217) | | (0.191) | | (0.581) | |  |
| Non-HBE |  | |  | |  | |  |  | |  | |  | |  |
| Inverse mills ratio(lambda) | -0.057 | | -13.164*** | | 0.854 | |  | 0.458 | | 0.009 | | 2.878 | |  |
|  | (0.749) | | (1.948) | | (1.046) | |  | (0.324) | | (0.115) | | (2.134) | |  |

Robust standard errors in parentheses; ***, ** and * significant at 1%, 5% and 10% respectively.

Source: Authors’ Computation using GHS Cross-Sectional Panel Data (2010 – 2015).
