# Supplementary material for: Participation and returns from informal service-oriented non-farm enterprises: Evidence from a survey of Nigerian households
Source: PLoS One. 2024 Mar 18;19(3):e0298794. doi: 10.1371/journal.pone.0298794 (PMC10947675; doi:10.1371/journal.pone.0298794)
Supplement: Attachment — Submitted filename: PONE-D-23-24888 - Comments.pdf [file pone.0298794.s003.pdf]

PLOS ONE

Participation and returns from informal service-oriented non-farm enterprises: Evidence from a survey of Nigerian households

--Manuscript Draft--

**Manuscript Number:**	PONE-D-23-24888
**Article Type:**	Research Article
**Full Title:**	Participation and returns from informal service-oriented non-farm enterprises: Evidence from a survey of Nigerian households
**Short Title:**	Informal service-oriented non-farm household enterprises and earnings in Nigeria
**Corresponding Author:**	Ikechukwu Darlington Nwaka, Ph.D<br>University of Alberta<br>Edmonton, AB CANADA
**Keywords:**	Informal Service Sector, Non-Farm Enterprise, Home-Based, Non-Home-Based, Random Effects, Probit, Nigeria.
**Abstract:**	We investigate the determinants of selection and productivity of informal service-oriented family enterprises in Nigeria. Using national representative micro-data from the Nigerian General Household Survey (2010-2015), random effect probit and selectivity adjusted regression models were estimated and the results examined. Findings show that the location of informal Non-Farm Household Enterprise (NFHE)s (home-based or non-home-based), matter considerably in the wholesale and retail service sectors as well as in the personal and consultancy service sectors operated by informal NFHEs. This result remained the same even after controlling for heterogeneity across individuals, households or locational characteristics. Similarly, by controlling for selectivity in the earnings equation, it was observed that home-based informal enterprises are less productive compared to non-home-based ones and which varies across sectors. Overall, the gender of business owners and educational levels, as well as geopolitical zones, infrastructure and business characteristics do matter in the locational and productivity differences of service-oriented enterprises in Nigeria.
**Order of Authors:**	Ikechukwu Darlington Nwaka, Ph.D
	Okechukwu Lawrence Emeagwali, PhD
**Additional Information:**	
**Question**	**Response**
**Financial Disclosure**<br><br>Enter a financial disclosure statement that describes the sources of funding for the work included in this submission. Review the submission guidelines for detailed requirements. View published research articles from *PLOS ONE* for specific examples.<br><br>This statement is required for submission and **will appear in the published article** if the submission is accepted. Please make sure it is accurate.	The funders had no role in study design, data collection and analysis, decision to publish, or preparation of the manuscript.

Open Rubric

Powered by Editorial Manager® and ProduXion Manager® from Aries Systems Corporation

**Unfunded studies**<br>Enter: *The author(s) received no specific funding for this work.*<br><br>**Funded studies**<br>Enter a statement with the following details:<br>• Initials of the authors who received each award<br>• Grant numbers awarded to each author<br>• The full name of each funder<br>• URL of each funder website<br>• Did the sponsors or funders play any role in the study design, data collection and analysis, decision to publish, or preparation of the manuscript?<br>• **NO** - Include this sentence at the end of your statement: *The funders had no role in study design, data collection and analysis, decision to publish, or preparation of the manuscript.*<br>• **YES** - Specify the role(s) played.<br><br>* typeset	
**Competing Interests**<br><br>Use the instructions below to enter a competing interest statement for this submission. On behalf of all authors, disclose any competing interests that could be perceived to bias this work—acknowledging all financial support and any other relevant financial or non-financial competing interests.<br><br>This statement is required for submission and **will appear in the published article** if the submission is accepted. Please make sure it is accurate and that any funding sources listed in your Funding Information later in the submission form are also declared in your Financial Disclosure statement.<br><br>View published research articles from *PLOS ONE* for specific examples.	The authors have declared that no competing interests exist.

Powered by Editorial Manager® and ProduXion Manager® from Aries Systems Corporation

**NO authors have competing interests**<br><br>Enter: *The authors have declared that no competing interests exist.*<br><br>**Authors with competing interests**<br><br>Enter competing interest details beginning with this statement:<br><br>*I have read the journal's policy and the authors of this manuscript have the following competing interests: [insert competing interests here]*<br><br>* typeset	
**Ethics Statement**<br><br>Enter an ethics statement for this submission. This statement is required if the study involved:<br><br>• Human participants<br>• Human specimens or tissue<br>• Vertebrate animals or cephalopods<br>• Vertebrate embryos or tissues<br>• Field research<br><br>Write "N/A" if the submission does not require an ethics statement.<br><br>General guidance is provided below. Consult the submission guidelines for detailed instructions. **Make sure that all information entered here is included in the Methods section of the manuscript.**	N/A

**Format for specific study types**<br><br>**Human Subject Research (involving human participants and/or tissue)**<br>• Give the name of the institutional review board or ethics committee that approved the study<br>• Include the approval number and/or a statement indicating approval of this research<br>• Indicate the form of consent obtained (written/oral) or the reason that consent was not obtained (e.g. the data were analyzed anonymously)<br><br>**Animal Research (involving vertebrate animals, embryos or tissues)**<br>• Provide the name of the Institutional Animal Care and Use Committee (IACUC) or other relevant ethics board that reviewed the study protocol, and indicate whether they approved this research or granted a formal waiver of ethical approval<br>• Include an approval number if one was obtained<br>• If the study involved *non-human primates*, add *additional details* about animal welfare and steps taken to ameliorate suffering<br>• If anesthesia, euthanasia, or any kind of animal sacrifice is part of the study, include briefly which substances and/or methods were applied<br><br>**Field Research**<br><br>Include the following details if this study involves the collection of plant, animal, or other materials from a natural setting:<br>• Field permit number<br>• Name of the institution or relevant body that granted permission	
**Data Availability**<br><br>Authors are required to make all data underlying the findings described fully available, without restriction, and from the time of publication. PLOS allows rare exceptions to address legal and ethical concerns. See the PLOS Data Policy and FAQ for detailed information.	No - some restrictions will apply

Powered by Editorial Manager® and ProduXion Manager® from Aries Systems Corporation

A Data Availability Statement describing where the data can be found is required at submission. Your answers to this question constitute the Data Availability Statement and **will be published in the article**, if accepted.<br><br>**Important:** Stating 'data available on request from the author' is not sufficient. If your data are only available upon request, select 'No' for the first question and explain your exceptional situation in the text box.<br><br>Do the authors confirm that all data underlying the findings described in their manuscript are fully available without restriction?	
**Describe where the data may be found in full sentences. If you are copying our sample text, replace any instances of XXX with the appropriate details.**<br><br>• If the data are **held or will be held in a public repository**, include URLs, accession numbers or DOIs. If this information will only be available after acceptance, indicate this by ticking the box below. For example: *All XXX files are available from the XXX database (accession number(s) XXX, XXX.).*<br>• If the data are all contained **within the manuscript and/or Supporting Information files**, enter the following: *All relevant data are within the manuscript and its Supporting Information files.*<br>• If neither of these applies but you are able to provide **details of access elsewhere**, with or without limitations, please do so. For example:<br><br>*Data cannot be shared publicly because of [XXX]. Data are available from the XXX Institutional Data Access / Ethics Committee (contact via XXX) for researchers who meet the criteria for access to confidential data.*<br><br>*The data underlying the results presented in the study are available from (include the name of the third party*	https://microdata.worldbank.org/index.php/catalog/1002

Powered by Editorial Manager® and ProduXion Manager® from Aries Systems Corporation

and contact information or URL).<br>• This text is appropriate if the data are owned by a third party and authors do not have permission to share the data.<br><br>* typeset	
Additional data availability information:	

Manuscript Click here to access/download;Manuscript;Manuscript.docx

Participation and returns from informal service-oriented non-farm enterprises: Evidence from a survey of Nigerian households

Ikechukwu Darlington Nwaka[1]* and Okechukwu Lawrence Emeagwali[2]

[1]Alberta School of Business, University of Alberta, Edmonton - Canada

[2]Department of Business Management, Girne American University

Kyrenia, North Cyprus, via Mersin 10, Turkey.

lawrenceemeagwali@gau.edu.tr

*E-mail: ike.nwaka@ualberta.ca ; ikenwaka@gmail.com
(Corresponding author)
ORCID: 0000-0002-4903-3976

Abstract

We investigate the determinants of selection and productivity of informal service-oriented family enterprises in Nigeria. Using national representative micro-data from the Nigerian General Household Survey (2010-2015), random effect probit and selectivity adjusted regression models were estimated and the results examined. Findings show that the location of informal Non-Farm Household Enterprise (NFHE)s (home-based or non-home-based), matter considerably in the wholesale and retail service sectors as well as in the personal and consultancy service sectors operated by informal NFHEs. This result remained the same even after controlling for heterogeneity across individuals, households or locational characteristics. Similarly, by controlling for selectivity in the earnings equation, it was observed that home-based informal enterprises are less productive compared to non-home-based ones and which varies across sectors. Overall, the gender of business owners and educational levels, as well as geopolitical zones, infrastructure and business characteristics do matter in the locational and productivity differences of service-oriented enterprises in Nigeria.

Keywords: Informal Service Sector, Non-Farm Enterprise, Home-Based, Non-Home-Based, Random Effects, Probit, Nigeria.

1. Introduction

How do the characteristics of households affect the participation and productivity of informal Non-Farm Household Enterprises (NFHEs)? This question resonates with the complicated nature of the informal economy in Nigeria and other sub-Saharan African (SSA) countries. Between 2010 and 2015, the Nigerian General Household Survey panel data reported that 94% of all household enterprises are informal – conceptualised as those that are not officially registered with the government[1]. It also reported that 48% (see Table 1) of employment in such informal businesses operated outside the household's environment (i.e. non-Home-Based Enterprises (non-HBEs - henceforth)) in contrast with the other 52% which operated within the household's environment (Home Based Enterprises (HBEs - henceforth)). In the literature three factors distinguish the HBS from non-HBS. First, relates to limited business start-up costs amongst the HBEs which easily gets financed from owner's personal savings without paying the business space (Vorley & Rodgers, 2014; Daniel, Di-Dominico, & Sharm, 2015) unlike the non-HBEs that requires such payments including other financial obligations (Darja, 2016). Second, the time-use cost is greatly minimized for the HBEs than the non-HBEs resulting in flexible outcomes in value creation between household and labour market productions equally. Such reduced time-used costs refers to relative ease – like lower transport costs from home to work including the relative ease in balancing ecomomic and household production especially amongst women (Walker, 2004; Thompson, Jones-Evans, & Kwong, 2009; Nwaka, Guven-Lisaniler, & Tuna, 2016). Third, HBEs offers what Lipton (1980) call fungible consumption patterns the household. Lipton (1980) describes the household enterprises as fungible; that is the ability to swiftly convert available business resources into alternative uses. These differences suggest that factors affecting choices of service-oriented enterprises might vary across location with associated differences in productivities by location (HBEs and non-HBEs).

Descriptive data from the World Bank's Development Indicators (2015) provides much evidence of the value added to the Nigerian economy by its services sector. For instance, the agricultural sector, which contributed about 26% to its GDP and 57% to its employment numbers in year 2000, dropped considerably to 20% and 36% respectively in 2015. Within the same period, its service sector gained prominence, with contributions to GDP more than doubling from 22% in year 2000 to about 59% in 2015, mostly as a result of the 2014 rebasing of the nation's GDP; simultaneously, its contributions to employment numbers rose exponentially from 33% in year 2000 to almost 52% as of 2015 (World bank, 2015). Such burgeoning contributions of the service sector to the Nigerian economy are driven mostly by the effects of structural reforms and diversification policies away from agriculture and oil into other productive sectors (Fajana 2008). This has made the country's economy one seemingly led by the service sector. With this growing relevance of the service sector, informal enterprises have similarly burgeoned where the wholesale and retail trade accounted for most (about 53%) of the employment provided by these household enterprises within

[1] Our definition of informality in this paper is nonfarm household enterprises that are not officially registered with the government as reported from the survey.

the period under study. In terms of productivity as measured by earnings per worker, the average labour productivity in non-HBEs is 28,872 Naira/month (with median earnings totalling 8916 Naira per month), while that of HBEs was 19,620 Naira/month (with a median value of 3,658 Naira per month). The huge productivity differences between the mean and median values recorded above is as a result of the disparate characteristics of household enterprises. In particular, HBEs or non-HBEs employing more than one staff member typically record larger productivity levels as opposed to sole proprietorships or those who employed only one staff. In this case, the latter outnumbers the former hence the apparent disparities in mean and median values for both HBEs and non-HBEs. This corroborates the findings of Fox & Sohnesen (2012), in which they examined the quality of growth in Africa. Thus, we can glean from the descriptive analysis that productivity of non-HBEs is almost 47% higher than that of HBEs. Considering these differences, value added per worker is quite marginal to contribute to a decent standard of living for the households involved. Such differences in terms of sectoral composition and productivity present cases of fungibility due to the location of such enterprises, especially HBEs. Lipton (1980) describes the household enterprises as fungible; that is the ability to swiftly convert available business resources into alternative uses.

Even though these enterprises contribute significantly to employment and welfare, little is known about the antecedents and determinants of service operation in Nigeria. This study therefore explores two fundamental questions:

- What are the determinants of participation and productivity within the Nigerian informal service-oriented (wholesale and retail trade versus personal and consultancy services) NFHEs?
- Do the determinants of productivity in such enterprises differ based on location (HBE and Non-HBEs)?

Table 1: Here

Relying on NFHEs as a unit in our analysis, studies on non-farm enterprises in several developing countries are substantial (Naglar and Naudé, 2014; Oostendorp, Trung and Tung, 2009; Haggblade, Hazell and Reardon, 2007; Haggblade and Hazell, 2001; Berdegue, Ramirez and Rearson, 2001; Corral and Reardon, 2001). These, amongst other studies are limited to rural non-farm enterprises without a clear-cut demarcation of the level of formality or informality. In Africa, related studies on informal NFHEs are those of Livingstone (1991), Haggblade and Brown (1989), Moock, Musgrove and Stelcner (1989) amongst others. Again, these are not specific to sector, service orientation or location (HBE and non-HBE). Studies on HBEs versus non-HBEs include Tipple (2006 and 2005), Kellett and Tipple (2000) and Lipton (1980). These streams of studies are however not empirically grounded which are linked to developing countries of Asia. As such, they did not specifically analyse the sectoral domain of such enterprises. From the forgoing and to the best of our knowledge, no study has been advanced to examine the determinants of participation and productivity among informal wholesale retail trade and personal/consultancy oriented family

businesses in SSA and Nigeria in particular, let alone the impact of location (whether HBEs or non-HBEs). This paper is, therefore, intended to update the literature by offering empirical support by means of a probit model and selectivity corrected Mincerian model to observe individual, household or geopolitical attributes that explain the probability of operating informal service-oriented non-farm enterprises in Nigeria. Besides this, the research also raises policy concerns from such findings.

Using the General Household Survey Cross-Sectional Panel Data (2010 – 2015), the study finds that the location of informal NFHEs being HBE or non-HBE matter considerably in both the wholesale/retail and personal service-oriented enterprises even after controlling for heterogeneity across individuals, households or locational characteristics. Similarly, by controlling for selectivity in the earning equation, it was observed that home-based informal enterprises are less productive compared to the non-home-based, which also varies across the wholesale/retail and personal service-oriented enterprises. For the overall joint model, personal and consultancy service-oriented enterprises and/or HBEs show a strong incidence of lower value added per worker compared to the wholesale, retail trade and/or non-HBEs. This further justifies heterogeneity incidences of the informal economy.

This paper is organized as follows: Section 2 reviews the nature of informal service-oriented enterprises in Nigeria, while section 3 handles data and variable definitions. In sections 4 and 5, we present the research methods and empirical findings. Finally, section 6 draws the conclusion and presents policy recommendations.

2.1 The Context: Informal Sectors

Terminology used in the description of the informal sector has evolved over the years with various schools of thought arising in the process. According to Williams (2008) who has extensively reviewed conceptualisations of informality and whose work has profoundly contributed in shaping contemporary thinking about the informal sector, introducing some of the most prominent terminologies used to describe the sector include cash-in-hand, undeclared, hidden, black, shadow, and underground among others. While these terminologies appear to point to the same underlying concept, three major terminologies have found profound usage in the labour literature. These three include ‘informal’, ‘undeclared and ‘shadow’ economy (Hart, 1985; Sassen, 1993; Chen, 2012; William, 2008, William and Horodnic 2015, Schneider and Enste, 2000, Schneider, 2012, Schneider and Buehn, 2018, Buehn, Dell’Anno and Schneider, 2018, Enste, 2018, Berdiev and Saunoris, 2018; Webb et al., 2009; ILO, 2013). Irrespective of the seemingly disparate terminologies and definitions, one can see that they all allude to the same underlying thread: economic activities outside the purview of the state with no tax obligations and similarly without government protection and aid.

This study chooses to use the term ‘informal sector’ as put forward in Williams, (2009) because its all-encompassing nature well suits the nature of the data, we are dependent on (household data).

However, in light of the three major categorical activities within this sector, as mentioned by Williams, (2009), our conceptualisation of the informal sector falls squarely within the 'paid informal work' category. Furthermore, the definition of our conceptualisation of informal enterprises falls within, and is most appropriately captured in, the definition put forward by the Fifteenth International Conference of Labour Statisticians (15th ICLS) in 1993 (ILO, 2000) which defines informal enterprise on the following basis, that such an enterprise:

- is unincorporated, owned and managed by a member of the household
- is market oriented, producing goods and services for sale.
- employs a given number of employees or own account worker.
- is non-agriculturally oriented which is not officially registered with the government.

Informal employments are thus seen as "a survival activity of the very poor" (Sudarshan and Unni,2003). Diverse views on the causes, composition, and nature of informal economy[2] exist. According to the ILO (1972), the dualists see the informal economy as unregulated and small autonomous family orientated businesses. The structuralists, on the other hand (Castells and Portes, 1989) see the sector as heterogeneous due to the actions of formal capitalist firms in response to structural changes. In another vein, the legalists (De Soto,1989), ascribe the sector to have emanated due to some legal and governmental bureaucratic formalisation processes. The voluntarists (Maloney, 2004) however, hold that the informal sector exists by choice. Informal economies play a fundamental role in the economy of nations. However, there appears to be no consensus on its exact role (Fethi et al. 2006). One of the most prevalent theories on the consequence of the informal sector on economies is that of the loss of tax revenues accruable to the state (Fethi et al. 2004; **Besim et al, 2015**). Though these perspectives may be valid in certain circumstances, the informal sector is far more heterogeneous and complicated than already written (Chen, 2012). Within the NFHE literature, conventional wisdom holds that a significant proportion of such enterprises are more of a service led employment. Fox and Sohnesen (2012) assert that informal non-farm enterprises constitute vital sources of livelihood and economic development through employment generation of about 70%. This is attributed to the World Bank's review of household enterprises in SSA (World Bank, 2016). In the literature, the term non-farm enterprises connotes to informal household enterprises, with heterogeneity of activities comprising of agribusiness, wholesale and retail trade, construction, utilities, commerce, tourism and other services outside the farm (agriculture) (Nagler and Naudé, (2014); Haggblade, Hazell and Reardon (2010); Reardon (1997)).

2.2 Home-Based and non-Home-Based Service Oriented Enterprises

[2] See Williams (2009) and Chen(2012) for a comprehensive view on the characteristics of the informal sector. Particularly, disparate articulations are based on four distinct theses propositions are used to delineate the informal sector as documented by Williams (2009); the leftover thesis, reinforcement thesis, chosen alternative thesis and the involuntarily decanted thesis.

In the literature, decision to locate informal enterprises within the home (HBEs) and away from the home premises (non-HBEs) comes from three distinct factors. The first is tied closely to the cost-minimization hypothesis such that HBEs business start-up finances are ably sourced from owner's personal savings due to the available business space within the home premises unlike the non-HBEs that requires more capital for start-ups which partly is associated with cost of business spaces in several industrial locations or open spaces far from the home (Vorley & Rodgers, 2014; Daniel, Di-Dominico, & Sharm, 2015; Darja, 2016). Second, the time-use cost is greatly minimized for the HBEs than the non-HBEs resulting in flexible outcomes in value creation between household and labour market productions equally. Such reduced time-used costs refers to relative ease – like lower transport costs from home to work including the relative ease in balancing ecomomic and household production especially amongst women (Thompson, Jones-Evans, & Kwong, 2009; Meisam, Zahra, Kambiz, & Maghsoud, 2017). This particularlily household production rests oon child-care activities while also running HBEs. The literature also points to possible reduction productvitiy efficiency of HBEs due to inherent managerial lapses a(Williams, 2004; Joona, 2018) which however is different for the onsite non-HBEs where posductivity is much more. Third, HBEs offers what Lipton (1980) call fungible consumption patterns the household. Lipton (1980) describes the household enterprises as fungible; that is the ability to swiftly convert available business resources into alternative uses (Kellet & Tipple, 2000). Earnings from informal sales are easily tranfered to domestic consumption or to the business which improves living standard of family members. This may also lead to possible losses due to negative spillover effects from such consumption patterns resulting in heterogeneity[3] due to location of such enterprises, whether HBEs or non-HBEs. Within the "extended fungibility" criterion, Lipton (1980) further opines that the HBEs can readily convert household resources into domestic consumption for food and health, or economic consumption from business expenses when compared to the non-HBEs.

These differences suggest that factors affecting choices of service-oriented enterprises might vary across location with associated differences in productivities by location (HBEs and non-HBEs). In general parlance, given that the wholesale and retail trade oriented businesses require open spaces in public or on-site locations to meet the demand for its services, it is therefore argued that non-HBE are more wholesale retail businesses oriented compared to those of HBEs (Vorley & Rodgers, 2014; Daniel, Di-Dominico, & Sharm, 2015; Lipton 1980). By extension, the composition of outputs from wholesale retail businesses require direct proximity to the market for its goods which ordinarily might be different for personal and consulting services that might require large pool of residential clienteles to petronize such services (Strassmann 1986).

2.3 Nature of Informal Service-Oriented Household Enterprises in Nigeria.

[3] Heterogeneous concept is loosely defined to imply different characterization of the informal nonfarm enterprises and differences in factors affecting its performance.

The Nigerian General Household Survey Panel Data generated various information and characteristics of nonfarm family business across 5000 households and their members. From these, over 95% of households operated at least one non-farm enterprise or income generating activity. Thus, analysis will be based on survey reports on enterprises that are non-farm (non-agricultural based) in nature. The data in Table 2 presents the distribution of informal service-oriented enterprises by geopolitical zones, region and gender, between 2010 -2015. As will be observed from Table 2, a higher concentration of HBEs and non-HBEs are found in the South-West region. This is likely as a result of the population density in Lagos, with its vast commercial and economic opportunities. HBEs, which are personal and consultancy service-oriented, are relatively more concentrated in the rural areas than the urban. For Non-HBEs, personal and consultancy services are also highly concentrated in the urban areas. Also, comparing HBEs and Non-HBEs according to owner's gender, it could be observed that female enterprise owners are more home-based. However, they are non-home-based only in the wholesale and retail trade-oriented enterprises. Male-owners on the other hand are seen to be highly represented in the Non-HBE personal and consultancy-oriented enterprises. This further suggests a possible gender difference in the operation of HBEs and non-HBEs in Nigeria.

Table 2: Here

Figure 1: Here

An important question for determination is thus: is post-secondary education a determinant for operating an HBE or Non-HBE? Taking a look at Figure 1, it is clear that primary and secondary education dominate the wholesale and retail-oriented enterprises (HBEs and Non-HBEs), while secondary education determines personal and consultancy-oriented enterprises.

Table 3: Here

Figure 2: Here

Another important question for determination is: do revenues from HBEs and Non-HBEs vary across the service-oriented enterprises and gender? Table 3 reports the sales revenue of HBEs and Non-HBES by enterprise activity and gender. The Table shows that mean sales revenue of HBEs is lower than those of Non-HBEs in the wholesale and retail enterprises. Conversely, in the consultancy-oriented enterprises, revenues from the HBEs are much higher than the Non-HBEs at mean. Considering the median revenue gap[4], Non-HBEs earn almost 55% and 60% more in the wholesale and retail trade enterprises respectively than the HBEs. For the HBEs only, revenue from wholesale and retail-oriented enterprises is 25% more than those of the consultancy and personal enterprises. In the non-HBEs, wholesale and retail enterprises earn 15% more than the

[4] The revenue gap is calculated at the median such ((Non-HBE- HBE)/Non-HBE)*100 or Revenues from Male-owned – Female-owned/Male-owned *100

consultancy and personal services. Additionally, median revenue from male-owned enterprises are larger in the non-HBEs with a revenue gap of 40%.

This is also similar for female owned enterprises where non-HBEs revenue is about 63% more than those of HBEs. Comparing gender differences in respect of revenue earnings, there is a higher gender revenue gap in the HBE (47%) than the non-HBEs (13%). This higher revenue gap in the HBEs is an indication of an increasing female role in the household other than running the enterprise. Also, Figure 2 reports the average labour productivity, profits, and expenses for HBEs and non-HBEs including gender. Mean profits is therefore highest in the consultancy and personal services for the HBEs than that of the non-HBEs. Also, business profits are much higher in male-owned enterprises.

The above analysis further points to potential heterogeneity in informal service-oriented enterprises, which is partly due to differences in concentration and productivity. This study therefore, further accounts for the other possible factors influencing such heterogeneity, putting into consideration households and enterprise characteristics. This would be the focus of the following sections.

3. Data and Variable Definitions

3.1.Data

The GHS panel data of the Nigerian Bureau of Statistics (NBS) covering three waves (2010/2011—wave 1, 2012/2013—wave 2 and 2014/2015—wave 3) was applied in the analysis. The data is a national representative of 5000 households drawn from the various regions of the country. The questionnaires adopted include the household and community questionnaires from the post-planting and post-harvest sections. The household section documents the demography, education, employments and non-farm enterprises, while the community counterpart documents the access to basic facilities and infrastructure. Conceptualizing the informal sector is derived from conventional definition. Thus, informal household enterprises as used in this work are enterprises that are not officially registered with the Corporate Affairs Commission (CAC). This is in harmony with the 15th ICLS definition earlier mentioned. In this study, we classify informal enterprises as those responding “NO” to the survey question “Is this enterprise/business officially registered with the government?” Similarly, service-oriented enterprise as used in this paper comprises the activities outlined in Appendix A, which are broadly categorised as Wholesale, Retail and Trade or Consultancy and Personal Services.

From this, our sample consists of 3255 households with non-farm enterprises. The overall sample is therefore made up of 5557 HBEs and 5377 non-HBEs from the three waves. The definition of variables and summary of statistics are presented in Tables 4 and 5. These tables present various determinants of service-oriented enterprises and productivities for HBEs and non-HBEs. Particularly, the mean differences across the wholesale retail trade and personal/consultancy services are provided to show if the means of the characteristics are different across sectors and by

business location (Table 5). In the HBEs, age groups (<25 and >55), education (primary), gender, geopolitical zones (North-Central, North-East and West or South-South), rural locations, electricity connection and productivity are statistically significantly different at means in the wholesale retail trade and personal/consultancy oriented enterprises. These factors variables are different which could account for possible sources of heterogeneity – where factors affecting the choices of HBE wholesale retail trade versus personal/consultancy oriented types are not the same in each sector. However, compared to the HBEs, the observed characteristics in the non-HBEs seem to be even more heterogeneous commanding significant mean differences across the wholesale retail and trade and personal/consultancy oriented businesses are different, resulting in possible disparity between the HBEs and non-HBEs. Several instances have pointed to heterogeneous nature of labour demand and supply in the Nigerian economy (Guven-Lisaniler et al 2018). Concurrently, labour and demographics, household, infrastructure and business characteristics are included either in the probit models and or earning equations as defined below.

Table 4: Here

Table 5: Here

3.2. Dependent Variables

This study will employ several dependent variables. First, we use a binary variable (wholesale and retail trade =1 versus consultancy and personal service= 0) in the probit model. The value added per worker (productivity) will be estimated in the second model using the pooled OLS and random effects estimates accordingly.

3.3 Independent Variables

Labour and Demographic Characteristics

The labour and demographics are used to capture informal household owners' heterogeneity as well as heterogeneity by geopolitical zones and regions. Consequently, the age categories capture the effects of varying age brackets on informal business ownership and productivity. It has been argued that youth of less than 25 years in SSA are less likely to own informal enterprises compared to older ages (Fox and Sohnesen, 2012). Educational levels[5] will capture the effects of human capital endowment. Table 5 shows that a large proportion of the HBEs and non-HBEs are of the Middle Age range (26 to 45 years) or below the post-secondary level of education. Nwaka et.al. (2016) researched on the possible effects of geopolitical differences in the heterogeneity of labour demand. Thus, the geopolitical zones and regions are used to control for such effects. The effects of gender in business ownership within the HBEs and non-HBEs have presented different patterns in the developing countries, where female household owners are more of HBEs than the males (Tipple, 2006). The gender of business owner will reflect the probabilities of male or female

[5] The categorical variable of educational level is used instead of the continuous years of schooling in order to control for potential measurement errors (Pham and Reilly, 2007; Nwaka et al 2016)

participation in any of the service-oriented enterprises within the HBEs and non-HBEs, including the associated productivity differences. From Table 5, male-owners make up a larger proportion of non-HBEs than the HBEs.

Household Characteristics

Households differ in terms of attributes and sizes. Thus, households of larger size, who own their place of residence or with the presence of paid and unpaid household member, will present varying impacts on business operations and productivity. These variables will feature in the probability and earning models, except the home-ownership variable. Importantly, home ownership may also present sources of financial capital, through the financial institutions which affects the probability of operating any of the informal businesses (Flaig, 2014).

Infrastructure and Business Characteristics

The availability of electricity, transportation facilities, and microfinance institutions can stimulate the operation of such enterprises. Infrastructural deficiency has been presented as some inhibiting factors militating against the growth of non-farm enterprises in the developing countries (Lambon-Quayefio, 2017; World Bank, 2008). Also, we include the turnover ratio measuring the efficiency and ability of enterprises to recoup sufficient sales revenue due to its assets (Scarborough 2014). A high turnover ratio will show an indication of sufficient revenue from sales relative to assets. Table 5 also shows that turnover in the non-HBEs is higher than those of HBEs across the industries.

From the foregoing, home-ownership, paid and unpaid household members, and enterprise profits are the exclusion restriction variables. These variables will feature in the probit model and from the earnings equation.

4. Empirical Model

Central to the stated research questions, the first part of the study established the probability of having a wholesale/retail trade or consultancy/personal service-oriented informal enterprises, using a pooled panel and random affects probit framework. In order to guarantee an unbiased estimate of the productivity model, predicted probabilities from the probit model will be applied to the second stage of the model. This is necessary to check for potential selectivity bias in the main substantive model[6] of interest (Lee, 1973; Heckman, 1979).

In this study, we examine informal non-farm enterprises in Nigeria, dividing them into two categories: home-based (HBEs) and non-home-based (non-HBEs). These enterprises are assumed to be either engaged in wholesale and retail services or personal/consultancy services, based on data categorizations. However, our research solely focuses on service-oriented enterprises, excluding manufacturing-oriented ones. This allows us to exclusively analyze the unique

[6] This is akin to Mincerian wage equation of Mincer (1978)

characteristics and attributes of these service-oriented enterprises. Productivity model (earnings equation-henceforth) in each mode are determined as:

$$lnProd_{sit} = X_{sit}\beta_s + \mu_{sit} \;\; for\ each\ HBEs\ and\ non - HBEs \qquad (1)$$

where S = 1 for wholesale and retail service-oriented sector or S = 1 for consultancy and personal service-oriented sectors. $lnProd_{si}$ is the value added per worker (average labour productivity) for a given enterprise i in time "t" (such that t = 2010, 2012 and 2015) for each service-oriented enterprise S. The vector of broad categorization[7] of variables describing enterprise characteristics, such as the labour and demographics, household, infrastructure and business characteristics are represented by X. The β is a vector capturing parameter to be estimated, while μ is a vector of random disturbance term with a zero mean. We assume that non-farm informal employees' sectoral choices are conditioned to be in any of S (wholesale and retail trade versus and persona/consultancy services oriented) based on utility from such venture (Gindling, 1991).

Thus,

$$O_i = MaxV_{si} \qquad (2)$$

where V is the employee's/business owner's unobserved utility derivable from each employment mode, and O_i is the associated benefit employees derived from S. By transforming (2) as a linear function of employees' or enterprise observed characteristics and unobserved heterogeneity among the various service-oriented workers and enterprises, we obtain:

$$V_{si} = U_i\gamma_s - \varepsilon_{si} \qquad (3)$$

Where, γ represents a vector of parameters, U_i is a vector of variables explaining sectoral choices while ε represents the random disturbance term having a zero mean. The probability of each enterprise being observed as a (wholesale/retail trade=1 versus consultancy and personal services=0) can be written as:

$$P[(V_i = S = 1\,)] = \Pr[S > 0] = \emptyset(y_i\delta + \omega_i) \qquad (4)$$

where, y_i are vectors of exogenous observed characteristics as shown in table 5, δ is the parameter vectors due to y, ω_i is the disturbance term while $\emptyset$ is the univariate probit function.

Equation (4) therefore, represents the pooled probit model which does not account for other time invariant unobserved heterogeneity in the panel structure. Thus, the study further implements the random effects probit model by controlling for the time-invariant unobservable in the disturbance term, such that $\omega_{sit} = \theta_s + \epsilon_{sit}$. Hence, θ_s captures the effects time invariant unobservable characteristics such as differences in informal service-oriented

[7] Broad categories are listed and defined in table 4.

classification or differences in owner's motivation in running any of the HBEs or non-HBEs (Maloney 2004; De Mel, McKenzie and Woodruff, 2010). Similarly, ϵ_{sit} captures other unobservables. The θ_s and ϵ_{sit} are normally distributed, independent and uncorrelated with any of the y_is. The random effects probit become:

$$P[(V_i = S = 1\ )] = \Pr[S > 0] = \emptyset(y_{it}\delta + \theta_i + \ \epsilon_{it}) \qquad (5)$$

The sectoral choices of informal non-farm employees in any of the service-oriented enterprises may be non-random, with a potential selectivity bias as a result of unobserved heterogeneity. Equation 1 as outlined above is valid when earnings from each NFHE are observed which represents a subset of the population. Thus, no selection bias is observed if this subset of the population is randomly selected. But, the decision to operate a given NFHE is often non-random – therefore requiring control for selectivity in the substantive model. Bearing this in mind, Lee (1983) further recommends a two-stage least squares to control for this. Thus, (1) becomes:

$$lnProd_{si} = X_{si}\beta_s + \lambda_{si}\delta_s + \eta_{si} \qquad (6)$$

where, $\lambda_{si} = \frac{\tau_{iS\theta}(\Theta^{-1}[P_{iS}])}{P_{iS}}$ implying the selectivity term or the inverse Mill's ratio[8] and η_{si} is the random element with zero mean. Equation (6) is consistent estimate of β_j if λs_i is significant, indicating presence of selectivity.

Similarly, controlling for time-invariant unobservable in the earnings equation, the study further takes advantage of the panel structure of the dataset. This is called for given that the pooled OLS of equations (1) and (6) may not account for such effects (Woodridge, 2002). The FE model provides consistent estimates only on the condition that the explanatory variables are time-invariant. The time-invariant attributes in our sample such as geopolitical zones, gender of the owner, are some important determinants of productivity that should not be ignored. To ably capture these characteristics, we apply a slight modification of the fixed effect model – Between Estimator (BE[9]) and the Random Effects (RE) model. The RE model is presented as:

$$lnProd_{sit} = X_{sit}\beta_s + \rho_{sit} \qquad (7)$$

where $\rho_{sit} = \alpha_{si} + \varphi_{sit}$ and α_{si} is the unobserved effect. Accordingly, the unobserved effects are treated as a random variable in the disturbance term. In the absence of any selectivity bias, equation (7) yields a consistent estimate over pooled OLS if α_{si} is distributed independently of all X_S.

5. Empirical Results

[8] Since employees are observed to be in any of the service oriented enterprises, the earning equation is however conditional on each sector, while the inverse mill ratios are generated from the predicted probabilities from equation (4).

[9] BE is a pooled OLS estimator applied to the means of dependent and explanatory variables while controlling for the unobserved fixed effects (see Woodridge, 2002).

5.1 Choice Models

We begin by analysing the probit models (pooled and random effects) as reported in Tables 6 and A2[10]. Thus, we present both the coefficients and the average marginal effects (AME[11]) estimates accordingly. Table 6 reports the full model by including HBEs as an explanatory variable. This is to observe the place of HBEs in determining service-oriented choices.

Table 6: Here

As will be observed, the different econometric results are sensitive to the approaches applied and the estimates of the pooled-probit and RE-probit show slight statistical differences across the joint model (Table 6) and or HBEs and non-HBEs (Table A2). Likelihood ratio test (chibar2) was used to discriminate between pooled-probit and RE-probit. Hence, the unobserved heterogeneity is highly significant which explains about 99% of unexplained variation across time and resulting in persistent level of heterogeneity in service-oriented choices based on time invariant unobservable. Therefore, using the RE-probit is basis of interpretation, location such as being HBEs or not, owner's age, educational level and gender, macro-regions, availability of electricity connections and presence of unpaid family member are significant determinants of informal wholesale retail and trade oriented service enterprises as a whole. Our results aligns well with studies for other developing countries where demographic attributes such as gender, age and education determines wholesale and retail trade operations (Ligthelm 2005; Ntema 2016). Particularly, HBEs have lower probability of being wholesale compared to the non-HBEs relative to consultancy-oriented sector. Thus, relying on the marginal effects estimates of Table 6, HBEs are less likely to be wholesale retail and trade oriented by about 3% points than enterprises specializing in consultancy and personal service businesses such as beautification, tailoring and laundering works. The literature holds that the driving force to HBEs rests on the ideals of cost-minimization and efficiency in time-use. This ably mirrors into the antecedents and diverse nature of home-based works in developing where most of such businesses thrive (Chen M. A., 2014). Since, wholesale retail and businesses require open spaces in public locations to meet the demand for its services, non-HBE wholesale retail businesses thrive more in such situations (Vorley & Rodgers, 2014; Daniel, Di-Dominico, & Sharm, 2015; Lipton 1980). By extension, the composition of outputs from wholesale retail businesses require direct proximity to the market for its goods which ordinarily might be different for personal and consulting services that might require large pool of residential clienteles to petronize such services (Strassmann 1986).

Since service oriented enterprises are heterogenous commanding differences locational attributes, Table A2 reports separate estimates amongst HBEs and non-HBEs for each service-oriented enterprise. Justification for having separate estimates of HBEs and non-HBEs is to see whether differences in observed and unobserved characteristics affect choices of service orientation differently. If this is the case, what are the impacts of these differences on

---

[10] See Appendix sections for the related tables.

[11] AME basically clarifies the degrees of change.

productivity? The interpretations are related wholesale retail and trade relative to the consultancy and personal-oriented service types. In both HBE and non HBEs, older household members above 55 years have a higher probability of being in the wholesale retail and trade sector than the younger members (< than 25). This is relative to operating a personal service and consultancy oriented businesses. This is very peculiar to similar observations in Africa. Thus, Fox and Sohnesen (2012)'s comprehensive study on household enterprises and livelihood in Africa shows that younger enterprise owners are less likely to operate informal businesses than the older ones due to difficulty in obtaining the required capital or experience towards business growth. Owners with post-secondary tertiary education are more inclined to being operating informal wholesale retail businesses than personal service types which is relatively highest amongst the non-HBE businesses

Also, male business owners have a lower probability of running wholesale and retail businesses compared to female owners, relative to personal and consultancy oriented types. This value is more negative in non-HBE (34.7.8% of Column 8) than the HBE (28.5% of Column 4). We can speculate that female owners' preference for home-based wholesale and retail trade enterprises is influenced by the flexibility criterion, allowing them to balance household responsibilities and labour market activities. (Thompson, Jones-Evans, & Kwong, 2009; Aridakis, Marlow, & Storey, 2014). The study reveals noticeable geopolitical disparities in the likelihood of operating home-based enterprises (HBEs) and non-home-based enterprises (non-HBEs) in wholesale and retail businesses. Comparatively, non-HBEs in wholesale and retail sectors show a higher probability of being in the North (East and West) and a lower probability of being in the South-West, in contrast to consultancy and personal service types. This finding indicates the presence of macro-regional variations in business locations within Nigeria. These observations align with previous research by Guven-Lisaniler et al. (2018), which also highlighted the prominence of commercial and other major activities in these geopolitical zones.

5.2 Productivity Estimates: HBEs and non-HBEs

Tables 7 and those in the Appendix (Tables A3 and A4) are the earnings estimates according to equations (1), (6) and (7). To discriminate between the pooled OLS and RE models, Breusch and Pegan Largrange Multiplier test (LM-Test) (Greene and McKenzie, 2012; Breusch and Pegan, 1980) was conducted. This confirms the consistency of RE over pooled-OLS models. Table 7 reports the full model (using HBEs and sector as determinants of productivity), while Tables A3[12] and A4 disaggregate the service-oriented enterprises by location (whether HBE or non-HBE).

Based on the choice models above, the probability of operating any of the wholesale and retail and/or consultancy and personal oriented enterprises varies by sector or location (HBEs and non-HBEs). For this reason, we have controlled for selectivity bias in the earnings equations (productivity estimates) by adding the inverse mills ratio (lambda) into the earnings models of (6)

[12] Please see the appendix section for related estimates.

and (7) accordingly. As already mentioned, the statistical (in)significance of the selectivity term confirms whether the determinants of service-oriented businesses and their locational characteristics has any impact on productivity other than the related exogenous variables (Gindling, 1991). Evidence from, columns (4, 6, 7, and 9) of Table 7; columns (1, 2 and 3) of Table A4 and column (2) of Table A4 show that that the selectivity terms are found significant using the t-statistic and insignificant for other columns not mentioned. The significant effects of the lambda terms imply possible selectivity bias in the affected models, which further necessitates their correction accordingly. In order to save space, we have therefore retained estimates where lambda is significant while also retaining the equations (1 and 7) for which it is insignificant. Thus, coefficients of the related selectivity terms are reported in the Appendix (Table A5).

Table 7: Here

Comparatively, across all Tables 7, A3 and A4, the coefficients of the RE models are considerably smaller in magnitude than the pooled OLS. These imply the significant effects of the time-invariant unobserved heterogeneity across the service-oriented enterprises. Thus, Table 7 indicates that the HBEs are less productive than the non-HBEs, thereby implying lower value added in both service-oriented enterprises. Compared to the both service oriented businesses, productivity is lowest in the wholesale retail trade sector (- 0.430) than that of the consultancy and personal service oriented types (- 0.332). This might be associated with Lipton (1980)'s fungible allocation hypothesis where cash emanating from sales are reallocated into households' consumption thereby leading to lower earnings from businesses. Other forms of "extended fungibility" rely on multiple use of time between running the business and engaging in other household productions. These consequently translate to lower value added for HBEs (Kellet & Tipple, 2000). Similarly, personal and consultancy-oriented enterprises command significantly lower productivity in all models. This therefore justifies the estimation of separate equations each for HBEs and non-HBEs across the service-oriented enterprises. To save space, inferences will be due to the RE models where possible. The BE-model is provided for robust checks.

Productivity and Informal Home-Based and non-Homebased Service Oriented Enterprises

As observed from Tables A3 and A4 owner's age, gender and educational level, geopolitical location, regional location, improvement in electricity generation, turnover, and the presence of paid family workers are determinants of productivity in the informal HBES and non-HBEs. Comparatively, across Tables A3 and A4, the coefficients of post-secondary education (wholesale retail and trade sector) under the OLS model is found negative which however changes to positive under the RE models – reflecting the effects of a possible heterogeneous random factors in the model. The Table A3 shows that most of the labour and demographic characteristics are statistically significant. According to Fox and Sohnesen (2012), education, gender and regional characteristics are the major determinants of informal enterprise earnings in Africa. We therefore will exclusively focus on these variables. For instance, except business owners with primary education, those with secondary and post-secondary certificates are more productive than the other

category, especially within the consultancy and personal service-oriented enterprises. This finding further supports that of Kuipie (2009); Fox and Sohnesen (2012); Nwaka et.al. (2016) and Guven-Lisaniler et.al. (2018) on the relevance of human capital on productivity. Male earnings premium is observed to be highest in HBE wholesale retail and trade sector (73.4% of the log of earnings) than the personal and consultancy oriented (58.7% of the log of earning). On the other hand, male earning premium are considerably larger in the non-HBE personal and consultancy oriented works (56% log of earnings) than the wholesale retail and trade sector (45.3% log of earnings). This finding resonates remarkably with that of Fox and Sohnesen (2012); Kweka and Fox (2011) where male earning premium amongst nonfarm business owners is considerably larger at about 65% log of earnings in Rwanda or 40% in Ghana and Tanzania. Reasons for such inequality by gender require further empirical scrutiny which falls outside the purview of this work. Across the macro-regions informal HB service-oriented enterprises within the North-East and North-West geopolitical zones are significantly less productive compared to similar informal enterprises in the North-Central. Due the economic opportunities prevalent in the South-West (Guven-Lisaniler et al 2018), informal HBEs within the wholesale and trade sector are more productive than those in the North-Central. We must comment on the relevance of infrastructure and institutions. Improvement in electricity generation, which is associated with rise in productivity within the wholesale and trade sector. In the consultancy and personal service-oriented sector, the mere presence of microfinance institutions is negatively associated with productivity. This may be related to the inability of such enterprises to secure loans for business growths from the microfinance firms, resulting in a negative effect on the productive outcomes Fox and Sohnesen (2012)

Conclusion

The question for determination in this study is whether location (Home-based and Non-Home-Based) does matter across several service-oriented industries. Having utilised the three waves of the General Household Survey (GHS) panel data set (2010/2011; 2012/2013; 2014/2015) to investigate the determinants of participation in service-oriented informal enterprises were analysed, putting into account what factors affect their productivity. After applying the Pooled and RE-probit, it was observed that the different econometric results are sensitive to the approaches applied where HBEs have lower probability of being wholesale or consultancy-oriented compared to the non-HBEs relative to personal/consultancy oriented types. After disaggregating enterprises by location (HBE versus non-HBE) and enterprise activity (wholesale, retail and trade or consultancy/personal service-oriented), the results are indicative of heterogeneity within the informal service oriented family enterprises and that the location of the businesses do matter in determining owners participation and earnings in Nigeria. We observed that location such as being HBEs or not, owner’s age, educational level and gender, macro-regions, availability of electricity connections and presence of unpaid family member are significant determinants of informal wholesale retail and trade oriented service enterprises as a whole which aligns well with studies

for other developing countries. Particularly, HBEs have lower probability of being wholesale compared to the non-HBEs relative to consultancy-oriented sector.

In terms of earnings (value added) HBEs are observed to be less productive than the non-HBEs which might be associated possible fungible resource allocation pattern from business venture into family consumption. Additionally, factors affecting productivity are equally different across the HBEs and non-HBEs in the service-oriented enterprises – which further confirm differential effects of business location in determining productivity among service led family enterprises in Nigeria. For the overall joint model, personal and consultancy service-oriented enterprises and/or HBEs show a strong incidence of lower added value per worker compared to the wholesale, retail trade and/or non-HBEs. Separate estimates for the HBEs and non-HBEs confirms that, owner's age, owner's educational level, owner's gender, geopolitical location of NFHE, regional location, improvement in electricity generation, turnover, and the presence of paid family workers all matter considerably in determining the productivity in at least one of the service-oriented NFHEs.

It is therefore safe to conclude that that factors affecting the choices of wholesale, retail and trade-oriented enterprises are different across location. Similarly, determinants of productivity within the HBE and non-HBE service-oriented enterprises varies in different magnitudes which further justifies heterogeneity incidences of the informal economy in the framework of this research. In other words, by studying the antecedents of NFHE operation and productivity in Nigeria, this study further recommends a massive improvement of employment and productivity potentials of the HBE and non-HBEs enterprise owners through several means.

First, since women are segregated and disadvantaged in participation and earnings, programmes that address these concerns are paramount. Secondly, financial inclusion should also be the prime focus of government policy which will allow for small business start-ups and economic growth. Third, reliance on the need to aggressively address infrastructural gaps through the provision of adequate electricity and good transport system. Finally, the informal sector is "here to stay", so policies that will champion the course of recognising the activities of the sector will be effective in solving the unemployment and poverty problems in Nigeria. Thus, given the burgeoning role of the service sector in employment generation in SSA, further studies exploring productivity differences among the formal and informal family non-farm enterprises across the region will further enrich the policy debates on the shadow economy. Furthermore, depending on feasibility, a replication of this study across the African continent is necessary to examine the generalisability of our findings across the four African regions. Finally, as with most quantitative analysis techniques, the Probit and Mincerian models deployed in this study focuses on examining the net effects of our predictor variables on the outcome variable.

More insight can be extracted if an alternative technique such as the combinatory approach using Fuzzy Sets Qualitative Comparative Analysis (FsQCA) is deployed. Therefore, we recommend that future researchers replicate this study using FsQCA or Latent Growth Curve Modelling (LGCM) analysis – a technique proven to be superior in the analysis of longitudinal data.

Disclosure of potential conflicts of interest

The authors declare that there are no competing interests.

Research involving human participants and/or animals

Not applicable.

Informed consent

Not applicable.

Availability of data and material

Not applicable.

Funding

Not applicable

References

Amin, A., Cameron, A. and Hudson, R. (2002), Placing the Social Economy, London: Routledge.

Alatas, V., & Newhouse, D. (2010). Indonesia jobs report: Toward better jobs and security for all. Washington, DC: Main Report, World Bank.

Andreß, H. J., Golsch, K., & Schmidt, A. W. (2013). *Applied panel data analysis for economic and social surveys*. Springer Science & Business Media

Berdiev, A. N., & Saunoris, J. W. (2018). Does globalisation affect the shadow economy? The World Economy, 41(1), 222-241.

Buehn, A., Dell'Anno, R., & Schneider, F. (2018). Exploring the dark side of tax policy: an analysis of the interactions between fiscal illusion and the shadow economy. Empirical Economics, 54(4), 1609-1630.

Breusch, T. S., & Pegan, A. R. (1980). The Lagrange multiplier test and its applications to model specification in econometrics. *The Review of Economic Studies*, *47*(1), 239-253.

Chen, M. A. (2012). The informal economy: Definitions, theories and policies. *Women in informal economy globalizing and organizing: WIEGO Working Paper*, *1*.

Castells, M., & Portes, A. (1989). World underneath: The origins, dynamics, and effects of the informal economy. *The informal economy: Studies in advanced and less developed countries*, *12*.

Davis, M. (2006), Planet of Slums, London: Verso.

De Soto, H. (1989). *The other path* (p. 17133). New York: Harper & Row.

De Soto, H. (2001), The Mystery of Capital: Why Capitalism Triumphs in the West and Fails Everywhere Else, London: Black Swan

Enste, D. H. (2018). The shadow economy in OECD and EU accession countries–empirical evidence for the influence of institutions, liberalization, taxation and regulation. In Size, Causes and Consequences of the Underground Economy (pp. 135-150). Routledge.

Fajana Sola (2008). "The Nigerian informal economy: Instigating decent work and pay, and national development through unionisation", Employee Relations, Vol. 30 Issue: 4, pp.372-390

Fethi, M. D., Fethi, S., and Katircioglu, S. (2006), Estimating the Size of the Cypriot Underground Economy: A Comparison with European Experience, International Journal of Manpower, 27 (6): 515 – 534.

Fethi, M. D., Fethi, S., & Katircioglu, S. (2004), Estimating the Underground Economy and Tax Evasion: Co integration and Causality Evidence in the Case of Cyprus, 1960-2003, International Journal of Economic Development, 6 (3): 120-157.

Fortin, B., Garneau, G., Lacroix, G., Lemieux, T. and Montmarquette, C. (1996), L'Economie Souterraine au Quebec: Mythes et Realites, Laval: Presses de l'Universite Laval.

Fox, L., & Sohnesen, T. P. (2012). Household enterprises in Sub-Saharan Africa: Why they matter for growth, jobs, and livelihoods: *World Bank Policy Research Working Paper 6184*

Fox, L. M., & Sekkel Gaal, M. (2008). *Working out of Poverty: Job creation and the quality of growth in Africa*. The World Bank.

Gindling, T. H. (1991). Labor market segmentation and the determination of wages in the public, private-formal, and informal sectors in San Jose, Costa Rica. *Economic Development and Cultural Change,* volume(issue), 585-605

Greene, W. H., & McKenzie, C. (2012). LM Tests for Random Effects

Guven-Lisaniler, F., Tuna, G., & Nwaka, I. D. (2018). Sectoral choices and wage differences among Nigerian public, private and self-employees. *International Journal of Manpower, 39*(1), 2-24.

Haggblade, S.; Hazell, P. and Reardon, T. (2007) *Transforming the Rural Non-Farm Economy: Opportunities and Threats in the Developing World*, International Food Policy Research Institute, Baltimore MD: Johns Hopkins University Press

Haggblade, S., Hazell, P., & Brown, J. (1989). Farm-nonfarm linkages in rural Sub-Saharan Africa. *World Development, 17*(8), 1173-1201.

Harris, J. R., & Todaro, M. P. (1970). Migration, unemployment and development: a two-sector analysis. The American economic review, 60(1), 126-142.

Hart, K. (1985). The informal economy. Cambridge Anthropology, 54-58.

Henderson, H. (1999), Beyond Globalisation: Shaping a Sustainable Global Economy, London: Kumarian Press.

Hudson, R. (2005), Economic Geographies: Circuits, Flows and Spaces, London: Sage.

International Labour Organization. (2013). Measuring informality: A statistical manual on the informal sector and informal employment. International Labour Office.

International Labour Office (2000): Resolution concerning statistics of employment in the informal sector, adopted by the Fifteenth International Conference of Labour Statisticians (January 1993); in: Current International Recommendations on Labour Statistics, 2000 Edition; International Labour Office, Geneva, 2000

International Labour Office. (1972). Employment, Incomes and Equality: A Strategy for Increasing Productive Employment in Kenya. Geneva: ILO

Lambon-Quayefio, Monica. Non-Farm Enterprises and the Rural Youth Employment Challenge in Ghana.**IDS Bulletin**, [S.l.], v. 48, n. 3, may 2017. ISSN 1759-5436. Available at: <http://bulletin.ids.ac.uk/idsbo/article/view/2873>. Date accessed: 07 aug. 2017. doi:http://dx.doi.org/10.19088/1968-2017.130.

Ligthelm, A. A. (2005). Informal retailing through home-based micro-enterprises: The role of spaza shops. *Development Southern Africa*, *22*(2), 199-214.

Loayza, N. V., & Rigolini, J. (2011). Informal employment: Safety net or growth engine? World Development, 39, 1503–1515.

Lee L (1983) Generalized econometric models with selectivity. *Econometrica* 51(2): 502–512.

Lewis, A. (1959), the Theory of Economic Growth, London: Allen and Unwin.

Livingstone, I. (1991). A reassessment of Kenya's rural and urban informal sector. *World development*, *19*(6), 651-670.

Maloney, W. F. (2004). Informality revisited. *World development*, *32*(7), 1159-1178.

Menkhoff T. & Seibel H. (1996) *Coping with Scarcity Microenterprise Strategies in Nigeria*, Sharbrucha GMH

McFadden, D. (1984). Econometric analysis of qualitative response models. In Griliches, Z. and Intriligator, M. eds. *Handbook of Econometrics, Vol. 2*. New York: Publisher, pp. 1395-1457.

Nagler, P. and Naudé, W. (2014) *Non-Farm Entrepreneurship in Rural Africa: Patterns and Determinants*, IZA Discussion Paper 8008, Bonn: Institute for the Study of Labor (IZA)

Nwaka, I. D., Guven-Lisaniler, F., & Tuna, G. (2016). Gender wage differences in Nigerian self and paid employment: Do marriage and children matter?. *The Economic and Labour Relations Review*, *27*(4), 490-510.

Ntema, J. (2016). Informal home-based entrepreneurs in South Africa'How non-South Africans outcompete South Africans'. *Africa Insight*, *46*(2), 44-59.

Reardon, T. (1997) 'Using Evidence of Household Income Diversification to Inform Study of the Rural Non-Farm Labour Market in Africa', *World Development* 25.5: 735–47 (accessed 22 February 2017)

Rothenberg, A. D., Gaduh, A., Burger, N. E., Chazali, C., Tjandraningsih, I., Radikun, R., ... & Weilant, S. (2016). Rethinking Indonesia's Informal Sector. *World Development, 80,* 96-113.
Sassen, S. (1993). The informal economy: Between new developments and old regulations. Yale LJ, 103, 2289.

Schneider, F., & Buehn, A. (2018). Shadow Economy: Estimation Methods, Problems, Results and Open questions. Open Economics, 1(1), 1-29.

Scarborough, N. M., (2014). Chapter 11: Creating a Successful Financial Plan. In *Essentials of entrepreneurship and small business management* (pp 407-427).England, Pearson Sudarshan, R. M., & Unni, J. (2003). Measuring the informal economy. *Informal Economy Centrestage. New Structures of Employment, Sage, New Delhi*, 19-38.

Strassmann, W. P. (1986). Types of neighbourhood and home-based enterprises: evidence from Lima, Peru. *Urban Studies*, *23*(6), 485-500.

Webb, J. W., Tihanyi, L., Ireland, R. D., & Sirmon, D. G. (2009). You say illegal, I say legitimate: Entrepreneurship in the informal economy. Academy of Management Review, 34(3), 492-510.

Williams, C. C. (2008), Cross-National Variations in Undeclared Work: Results From a Survey of 27 European Countries, International Journal of Economic Perspectives, Volume 2, Issue 2, pp. 46-63.

Williams, C. C. & Round, J. (2009), Out Of The Margins: Re-Theorizing The Role Of The Informal Economy In Ukraine, International Journal of Economic Perspectives, Volume 3, Issue 1, pp. 45-58.

Williams, C. C. (2010), Evaluating Competing Theories of the Shadow Economy: Some Lessons from an English Locality, International Journal of Economic Perspectives, Volume 4, Issue 2, pp. 391-402.

Williams, C. C. (2011), Blurring the formal/informal economy divide: beyond a dual economies approach, International Journal of Economic Perspectives, Volume 5, Issue 4, pp. 347-362
Williams, C. C., & Horodnic, I. A. (2015). Evaluating the prevalence of the undeclared economy in Central and Eastern Europe: an institutional asymmetry perspective. European Journal of Industrial Relations, 21(4), 389-406.
World bank (2016) Informal Enterprises in Kenya.; Worldbank Group, January

World Bank (2008) *World Development Report: Agriculture for Development*, Washington DC: World Bank

World Bank (2015) *World Development Indicators* Retrieved from http://data.worldbank.org/indicators| Spelling Errors

Figure Click here to access/download;Figure;TABLES AND FIGURES (1).docx

Table 1: Informal Nonfarm Household Enterprise Employments in Nigeria - Percentage Share and Productivity Differences (2010 -2015)

	**HBE**	**Non-HBE**	**OVERALL**
Formal	3	8	6
Informal	97	92	94
**Informal NFHEs**			
Overall Percentage Employment Share	51.53	48.47	100
**Sectoral Employment Shares**			
Wholesale and Retail Trade	49.70	58.55	53.99
Consultancy and Personal Services	19.21	23.72	21.40
Manufacturing	31.09	17.73	24.62
**Productivity**			
Average Labour Productivity/Month in Naira	19,630	28,872	24,092
Median Labour Productivity/Month in Naira	3,657	8,916	5,627

Source: Authors' Computation using GHS Cross-Sectional Panel Data (2010 – 2015).

Table 2: Weighted Distribution of Informal Service Oriented Enterprises by Geopolitical Zones, Region and Owner's gender (2010 – 2015)

	**Home Based**				**Non-Home Based**			
	Wholesale & Retail Number (in Millions)	%	Personal & Consultancy Number (in Millions)	%	Wholesale & Retail Number (in Millions)	%	Personal & Consultancy Number (in Millions)	%
**Zones**								
North-Central	2.701	11.19	0.777	8.74	3.954	15.01	1.196	10.49
North-East	3.329	13.80	1.529	17.21	2.110	8.01	0.654	5.73
North-West	5.886	24.39	2.579	29.02	3.409	12.94	1.327	11.64
South-East	1.388	5.75	0.450	5.06	4.405	16.73	1.591	13.95
South-South	3.902	16.17	0.939	10.56	4.440	16.86	1.294	11.35
South-West	6.922	28.69	2.614	29.41	8.017	30.44	5.341	46.84
**Region**								
Urban	10.543	43.70	3.451	38.83	12.671	48.11	6.811	59.73
Rural	13.584	56.30	5.437	61.17	13.664	51.89	4.593	40.27
**Gender**								
Female	19.157	79.40	5.369	60.41	16.145	61.31	3.000	26.30
Male	4.970	20.60	3.519	39.59	10.190	38.69	8.404	73.70

Source: Authors' Computation using the GHS Cross-Sectional Panel Data (2010 – 2015).

Figure 1: Distribution of Enterprises by Owner's Education

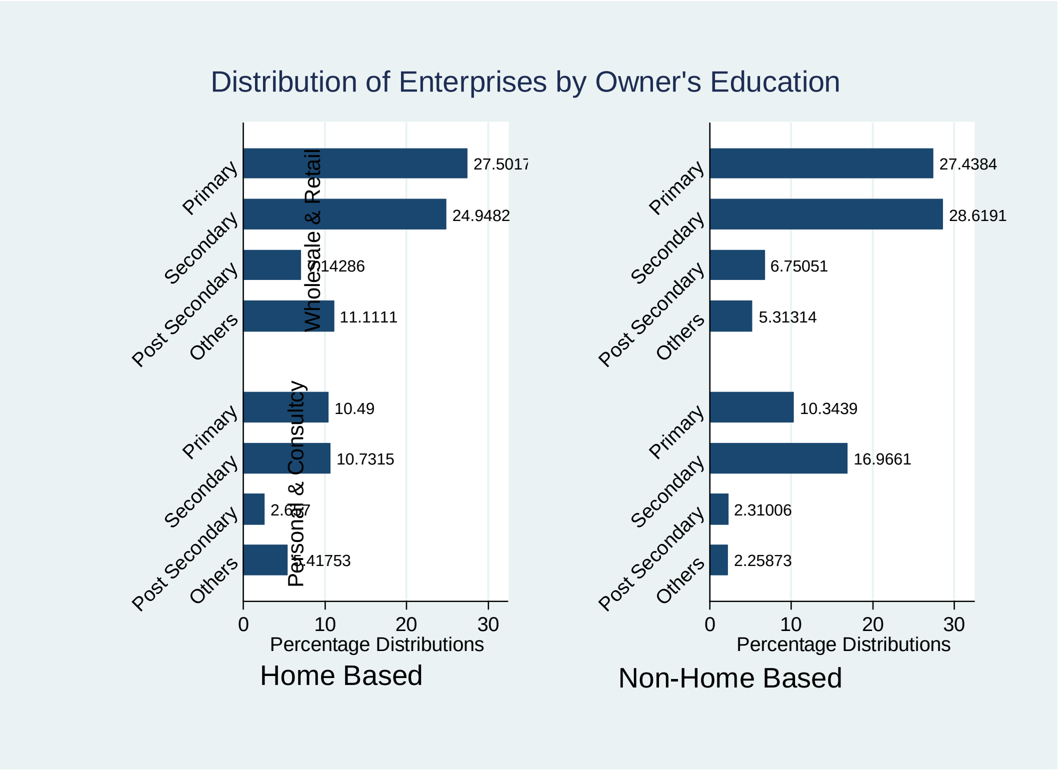

Table 3: Mean and Median Sales Revenue by (2010 and 2015), by Location, Service Sector and Gender.

	**Wholesale and Retail Trade (WRT)**	**Consultancy and Personal (CP)**	**Revenue Gap (Median %)**	**Male-Owned**	**Female - Owned**	**Gender-Ownership Gap**
**HBEs**						
Mean	26,263	141,465	25.4	119,373	13,805	46.7
Median	8,039	6,000		10288	5,486	
**Non-HBEs**						
Mean	66,784	72,096	15.2	92,621	31,049	12.5
Median	17,685	15,000		17,146	15,000	
**Median Revenue Gap (%)**	54.5	60		39.9	63.43	

Source: Authors' Computation using GHS Cross-Sectional Panel Data (2010 – 2015).

Figure 2:

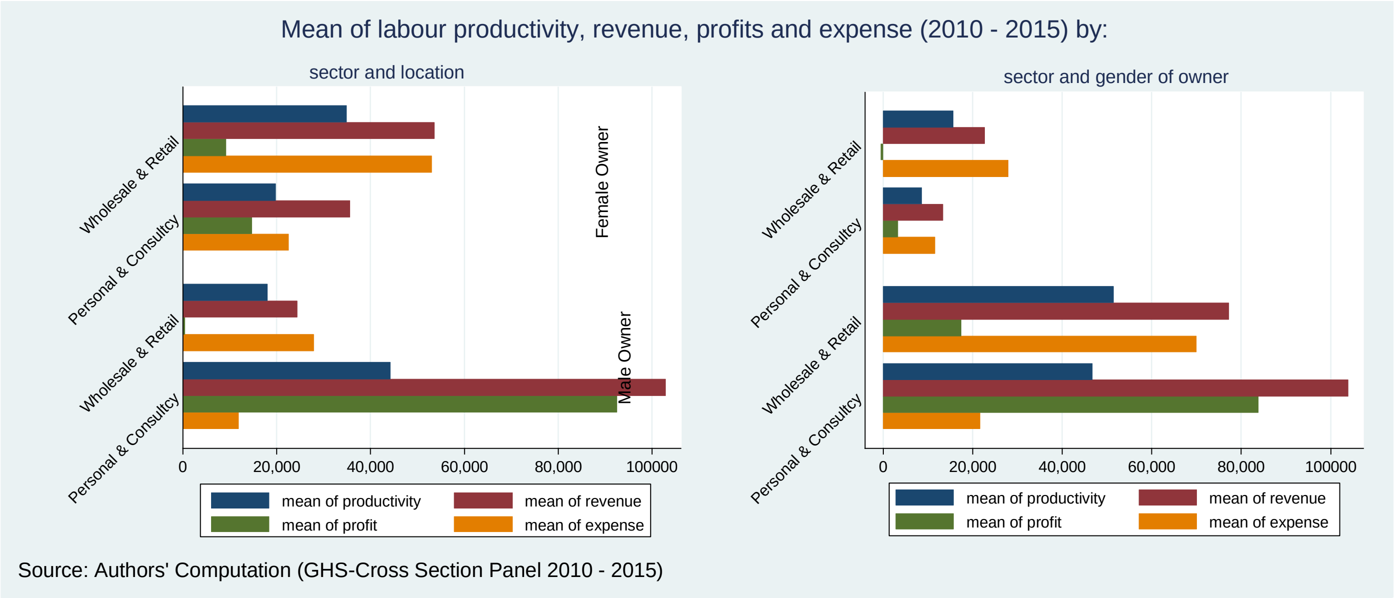

Table 4: Variable definitions

Variables	Variable Description
Dependent Variables	
**Probit Model**	

Informal Wholesale/Retail and Trade Services	=1 if is wholesale/retail and trade oriented which is not officially registered with the government and =0 if such businesses operates as nonfarm Consultancy and personal services oriented that is not officially registered with the government.
**Earnings Equation**	
Log of prod	Value added per worker= Total Revenue/No of workers
Independent Variables	
**Labour and Demographic**	Age of group of the family business owner (Base category=
**Characteristics**	=0 if between 15 – 25 years
Age Groups (Base <=25)	=1 if between 26 – 35 years
26 - 35	=1 if between 36 – 45 years
36 - 45	=1 if between 46 – 55 years
46 - 55	=1 if above 55 years
> 55	Level of education of the business owner relative to individuals categorized
Education (Base = Others)	(adult educated, Quoranic education or did not complete primary education)
	=1 if owner has primary education
Primary	=1 if owner completed secondary education
Secondary	=1 if owner has completed a post-secondary education
Post-Secondary	=1 if male owner, 0 otherwise
Gender_owner	Geopolitical zones (Base=North-Central)
Zones (Base= North-Cent)	=1 if located in the North-East
North-East	=1 if located in the North-West
North-West	=1 if located in the South-East
South-East	=1 if located in the South-South
South-South	=1 if located in the South-West
South-West	=1 if located in the rural areas, 0 otherwise.
Rural	
**Household Characteristics**	Number of household members
HH-size	=1 if owned residential homes, 0 otherwise
Own_home*	=1 if Business has a paid member of the household
Paid-HH*	=1 if Business has an unpaid member of the household
Unpaid-HH*	=1 if improvement in electricity connection in household, 0 otherwise
**Infrastructure**	=1 if improvement in electricity facility in the community, 0 otherwise
Electric	=1 if micro-finance institution is in the community, 0 otherwise
Transport	
Micro-finance	
**Business Characteristics**	
Profit*	Profit = Net revenues = Total sales revenue – Total costs
Turnover	Sales-to-Total-Assets Ratio=Sales/Total Assets

Note: * are excluded for the earnings equation (exclusion restriction). Note: Informality as used here reflects businesses that are not officially registered with the government at the time of the survey.

Table 5: Summary Statistics for the HBEs and non-HBEs Informal Service Oriented Enterprises.

	**HBEs**						**Non-HBEs**					
	Wholesale & Retail Trade		Consultancy & Personal		Mean Differences		Wholesale & Retail Trade		Personal & Consultancy		Mean Difference	
Variables	Mean	SD	Mean	SD	t-stat	p-value	Mean	SD	Mean	SD	t-stat	p-value
**Ages Categories**												
(Base Group<=25)	0.105	0.306	0.129	0.335	**3.112****	**0.002**	0.070	0.255	0.125	0.331	**6.166****	**0.000**
26 - 35	0.248	0.432	0.261	0.439	0.845	0.398	0.227	0.419	0.259	0.438	**2.045****	**0.041**
36 - 45	0.240	0.427	0.263	0.441	0.462	0.644	0.273	0.446	0.267	0.443	-1.878	0.060
46 - 55	0.164	0.370	0.167	0.373	0.243	0.808	0.201	0.401	0.174	0.380	-1.525	0.127
> 55	0.244	0.429	0.180	0.385	**-4.062****	**0.000**	0.228	0.419	0.174	0.380	**-2.925****	**0.004**
**Owner's Education**												
Primary	0.389	0.488	0.358	0.480	**2.098****	**0.036**	0.403	0.491	0.324	0.468	**-3.520****	**0.000**
Secondary	0.353	0.478	0.366	0.482	1.539	0.061	0.420	0.494	0.532	0.499	**5.296****	**0.000**
Post-Secondary	0.101	0.301	0.0907	0.287	-1.226	0.220	0.0991	0.299	0.0725	0.259	**-2.350****	**0.019**
Others=Base	0.157	0.364	0.185	0.388	1.710	0.085	0.0780	0.268	0.0709	0.257	-0.845	0.398
Male_owner	0.227	0.419	0.418	0.493	**13.515****	**0.000**	0.395	0.489	0.757	0.429	**22.888****	**0.000**
**Geopolitical zones**												
Zones (Base= North-Cent)	0.147	0.354	0.120	0.326	**-2.801****	**0.005**	0.206	0.404	0.148	0.355	**-4.202****	**0.000**
North-East	0.180	0.384	0.209	0.407	**4.128****	**0.000**	0.0903	0.287	0.0829	0.276	-0.571	0.568
North-West	0.258	0.437	0.305	0.460	**2.499****	**0.013**	0.131	0.338	0.130	0.336	-0.529	0.577
South-East	0.0622	0.242	0.0591	0.236	-0.539	0.589	0.184	0.387	0.161	0.368	**-2.064****	**0.039**
South-South	0.161	0.367	0.102	0.303	-3.879	0.000	0.168	0.374	0.137	0.344	**-2.305****	**0.021**
South-West	0.192	0.394	0.205	0.404	-0.639	0.523	0.221	0.415	0.342	0.474	**8.551****	**0.000**
Rural	0.653	0.476	0.685	0.465	**2.227****	**0.026**	0.606	0.489	0.521	0.500	**-5.287****	**0.000**
HH-size	8.139	4.073	8.207	3.900	0.659	0.510	7.308	3.374	6.810	3.368	**-3.532****	**0.000**
Own-Home	0.759	0.428	0.753	0.431	0.872	0.383	0.658	0.475	0.582	0.493	**-4.262****	**0.000**
Paid_hh	0.320	0.467	0.337	0.473	0.488	0.625	0.321	0.467	0.348	0.477	1.381	0.168
Unpaid_hh	0.308	0.462	0.284	0.451	-1.733	0.083	0.320	0.467	0.274	0.446	**-3.048****	**0.002**
electr	0.559	0.497	0.522	0.500	**-2.242****	**0.025**	0.606	0.489	0.640	0.480	**2.301****	**0.021**
com_trns	0.134	0.341	0.133	0.339	-0.089	0.929	0.145	0.352	0.127	0.333	-0.919	0.358
Microfin.	0.104	0.305	0.088	0.284	-1.684	0.092	0.811	0.273	0.994	0.300	**2.028****	**0.042**
Turnover	1.058	3.428	0.859	2.395	-1.408	0.159	2.262	34.91	2.357	30.73	-0.219	0.826
Profit	485.6	214,943	92,594	2.845e+06	1.701	0.089	9,237	802,360	14,760	86,797	0.054	0.957
Log Productivity	8.523	1.457	8.272	1.485	**-5.277****	**0.000**	9.308	1.372	9.047	1.320	**-5.265****	**0.000**
Observations	2772		1049				3176		1228			

Note: ***p<.05*; SD = Standard Deviation; t-stat=t-statistic

Table 6: Determinants of Service Oriented Household Enterprises using (non)Home-Based as explanatory variable (Pooled and RE-Probit Models)

	Wholesale and Retail Trade=1 vs Consult = 0			
	Pooled-Probit		RE-Probit	
VARIABLES	Coefficient	AME	Coefficient	AME
HBEs (non-HBE=0)	-0.205***	-0.069***	-0.288**	-0.026**
	(0.041)	(0.014)	(0.134)	(0.013)
Age Group (Base <=25)				
26 - 35	0.258***	0.096***	0.718***	0.090***
	(0.068)	(0.026)	(0.242)	(0.029)
36 - 45	0.448***	0.160***	1.372***	0.155***
	(0.068)	(0.025)	(0.258)	(0.029)
46 - 55	0.429***	0.154***	1.527***	0.166***
	(0.075)	(0.027)	(0.286)	(0.031)
> 55	0.619***	0.211***	2.008***	0.189***
	(0.077)	(0.027)	(0.308)	(0.030)
Education (Base = Others)				
Primary	0.197***	0.068***	0.588**	0.063**
	(0.074)	(0.026)	(0.256)	(0.030)
Secondary	0.148*	0.052*	0.527*	0.058*
	(0.076)	(0.027)	(0.272)	(0.032)
Post-Secondary	0.440***	0.142***	1.184***	0.102***
	(0.093)	(0.030)	(0.350)	(0.033)
Male_owner	-0.947***	-0.320***	-3.746***	-0.297***
	(0.042)	(0.014)	(0.263)	(0.014)
Zones (Base= North-Cent)				
North-East	0.196**	0.062**	0.694*	0.041*
	(0.085)	(0.026)	(0.376)	(0.024)
North-West	0.245***	0.076***	0.718**	0.042*
	(0.077)	(0.024)	(0.352)	(0.023)
South-East	0.023	0.008	0.016	0.001
	(0.073)	(0.024)	(0.329)	(0.028)
South-South	0.159**	0.051**	0.324	0.023
	(0.069)	(0.022)	(0.307)	(0.023)
South-West	-0.310***	-0.112***	-1.293***	-0.150***
	(0.068)	(0.024)	(0.321)	(0.033)
Rural	-0.024	-0.008	0.062	0.006
	(0.047)	(0.015)	(0.203)	(0.018)
HH-size	0.013**	0.004**	0.028	0.003
	(0.006)	(0.002)	(0.026)	(0.002)
Own_home	-0.004	-0.001	-0.106	-0.009
	(0.046)	(0.015)	(0.165)	(0.015)
Electric	-0.109**	-0.036**	-0.343**	-0.030**
	(0.047)	(0.015)	(0.159)	(0.013)
Transport	0.014	0.005	-0.028	-0.003

	(0.054)	(0.018)	(0.157)	(0.014)
Micro-finance	0.021	0.007	0.169	0.014
	(0.067)	(0.022)	(0.191)	(0.015)
Profit	-0.000	-0.000	-0.000	-0.000
	(0.000)	(0.000)	(0.000)	(0.000)
Paid_hh	0.063	0.021	0.136	0.012
	(0.042)	(0.014)	(0.117)	(0.010)
Unpaid_hh	0.120***	0.040***	0.243**	0.021**
	(0.042)	(0.013)	(0.114)	(0.010)
Constant	0.452***		2.838***	
	(0.124)		(0.488)	
Log Likelihood	-2928.36		-2374.14	
$\hat{\rho}$ (rho)			0.954	
			(0.003)	
Likelihood ratio			1108	
P-Value			0.000	
Observations	5,394	5,394	5,394	5,394
Number of id			3,363	

Robust standard errors in parentheses; ***, ** and * significant at 1%, 5% and 10% respectively. Note: AME=Average Marginal Effects.

Table 7: Determinants of Productivity Estimates for Informal HBEs and non-HBEs using (Pooled OLS, Between Effects and Random Effects Models)

	**Pooled OLS**			**RANDOM EFFECTS MODEL**		
	Wholesale and Retail Trade	Consultancy & Personal Services	Both	Wholesale and Retail Trade	Consultancy & Personal Services	Both
	1	2	3	7	8	9
C&P (WRT=0)			-0.442***			-0.396***
			(0.043)			(0.048)
HBE	-0.460***	-0.304***	-0.424***	-0.430***	-0.332***	-0.403***
	(0.048)	(0.085)	(0.041)	(0.049)	(0.079)	(0.041)
Age Group (Base <=25)						
26 – 35	0.196**	0.044	0.140**	0.159*	0.073	0.127*
	(0.078)	(0.117)	(0.066)	(0.084)	(0.112)	(0.068)
36 – 45	0.377***	0.195	0.322***	0.323***	0.188	0.279***
	(0.077)	(0.122)	(0.066)	(0.085)	(0.120)	(0.070)
46 – 55	0.435***	0.200	0.365***	0.398***	0.180	0.321***
	(0.086)	(0.140)	(0.073)	(0.094)	(0.133)	(0.077)
> 55	0.333***	0.126	0.270***	0.355***	0.125	0.292***
	(0.088)	(0.140)	(0.075)	(0.096)	(0.142)	(0.079)
Education (Base = Others)						
Primary	0.014	0.121	0.044	-0.014	0.182	0.025
	(0.084)	(0.127)	(0.070)	(0.088)	(0.136)	(0.074)
Secondary	0.293***	0.325**	0.303***	0.253***	0.367***	0.272***
	(0.089)	(0.138)	(0.075)	(0.092)	(0.141)	(0.078)
Post-Secondary	0.667***	0.694***	0.676***	0.629***	0.762***	0.646***
	(0.108)	(0.177)	(0.093)	(0.113)	(0.179)	(0.096)
Male_owner	0.648***	0.627***	0.625***	0.629***	0.611***	0.615***
	(0.053)	(0.082)	(0.043)	(0.059)	(0.085)	(0.049)
Zones (Base= North-Cent)						
North-East	-0.825***	-0.528***	-0.757***	-0.957***	-0.561***	-0.852***
	(0.107)	(0.171)	(0.090)	(0.120)	(0.176)	(0.102)
North-West	-0.366***	-0.538***	-0.412***	-0.570***	-0.526***	-0.566***
	(0.098)	(0.136)	(0.080)	(0.120)	(0.154)	(0.101)
South-East	-0.294***	0.002	-0.233***	-0.301***	-0.023	-0.224***
	(0.083)	(0.136)	(0.070)	(0.096)	(0.152)	(0.082)
South-South	0.244***	0.267**	0.245***	0.239***	0.316**	0.255***
	(0.080)	(0.131)	(0.068)	(0.089)	(0.143)	(0.076)
South-West	-0.174**	-0.238**	-0.205***	-0.172*	-0.234*	-0.188**
	(0.083)	(0.116)	(0.067)	(0.093)	(0.133)	(0.077)
Rural	-0.058	-0.120	-0.061	-0.035	-0.148	-0.049
	(0.053)	(0.093)	(0.046)	(0.062)	(0.097)	(0.054)
HH-size	-0.021***	-0.023*	-0.021***	-0.019**	-0.018	-0.019***
	(0.007)	(0.012)	(0.006)	(0.008)	(0.013)	(0.007)
Electric	0.192***	0.112	0.174***	0.150***	0.064	0.136***
	(0.054)	(0.092)	(0.046)	(0.055)	(0.089)	(0.047)
Transport	0.051	0.104	0.063	0.042	0.081	0.056
	(0.060)	(0.087)	(0.050)	(0.059)	(0.090)	(0.049)

Micro-finance	0.028	0.274**	0.093	-0.081	0.300***	0.024
	(0.081)	(0.134)	(0.070)	(0.076)	(0.111)	(0.063)
Turnover	0.004***	0.008	0.004***	0.004***	0.007	0.004***
	(0.000)	(0.007)	(0.000)	(0.001)	(0.005)	(0.001)
Paid_hh	-0.462***	-0.528***	-0.479***	-0.301***	-0.420***	-0.328***
	(0.049)	(0.075)	(0.041)	(0.045)	(0.069)	(0.038)
Inverse mills ratio (lambda)	**NS**	**NS**	**NS**	**S**	**NS**	**S**
Constant	9.021***	8.690***	9.058***	9.018***	8.649***	9.043***
	(0.139)	(0.218)	(0.118)	(0.152)	(0.236)	(0.129)
Observations	3,559	1,304	4,863	3,559	1,304	4,863
R-squared	0.220	0.238	0.225	0.217	0.235	0.223
Number of id				2,300	940	3,117
LM test Statistic				163.440	43.840	228.67
P-Value				0.000	0.000	0.000

Robust standard errors in parentheses. ***, ** and * significant at 1%, 5% and 10% respectively. C&P=Consultancy and Personal Services; WRT=Wholesale and Retail Trade; S implies that the selectivity term is significant in applied model, otherwise, NS (insignificant).

Appendix

Appendix A1: Description of Informal Non-Farm Household Enterprises

**Wholesale, Retail and Trade/ Repairs**
Wholesale and retail trade
Repairs of vehicles
**Personal and Consultancy**
Transportation services
Warehousing and support activities
Accommodation
Publishing activities
Motion picture, video, broadcasting and television programmes
Telecommunications, computer programming.
Financial, real estate and legal services activities.
Scientific research, advertising research a
Veterinary activities
Rental and leasing, travel agency, reservation
Education
Human health and social works activities
Residential care activities
Other personal service activities (barbers, launders etc)
Beauticians and tailoring

Source: Culled from the Nigerian GHS-Panel Data (NBS 2010 – 2015)

Table A2: Determinants of Wholesale and Retail Oriented Household Enterprises Home and Non-Home based (Pooled and RE-Probit Models)

	HBE				Non-HBE			
	Pooled-Probit		RE-Probit		Pooled-Probit		RE-Probit	
Variables	Coefficient	AME	Coefficient	AME	Coefficient	AME	Coefficient	AME
	1	2	3	4	5	6	7	8
Age Group (Base <=25)								
26 - 35	0.223**	0.080**	0.665*	0.076*	0.269***	0.102***	1.141***	0.126***
	(0.097)	(0.035)	(0.344)	(0.041)	(0.095)	(0.037)	(0.372)	(0.034)
36 - 45	0.298***	0.105***	0.853**	0.092**	0.516***	0.185***	1.949***	0.216***
	(0.099)	(0.036)	(0.369)	(0.043)	(0.094)	(0.035)	(0.392)	(0.031)
46 - 55	0.251**	0.089**	0.893**	0.095**	0.503***	0.181***	2.070***	0.226***
	(0.111)	(0.040)	(0.421)	(0.046)	(0.101)	(0.037)	(0.427)	(0.033)
> 55	0.556***	0.181***	1.979***	0.141***	0.609***	0.213***	2.400***	0.247***
	(0.113)	(0.037)	(0.467)	(0.043)	(0.107)	(0.038)	(0.458)	(0.031)
Education (Base = Others)								
Primary	0.221**	0.076**	0.488	0.046	0.068	0.023	0.529	0.061
	(0.097)	(0.035)	(0.354)	(0.037)	(0.113)	(0.038)	(0.404)	(0.050)
Secondary	0.232**	0.080**	0.578	0.053	-0.032	-0.011	0.211	0.026
	(0.102)	(0.036)	(0.387)	(0.039)	(0.115)	(0.040)	(0.417)	(0.052)
Post-Secondary	0.410***	0.134***	1.002**	0.076*	0.347**	0.106**	1.335**	0.119**
	(0.134)	(0.043)	(0.511)	(0.040)	(0.136)	(0.043)	(0.537)	(0.052)
Male_owner	-0.762***	-0.267***	-3.308***	-0.285***	-1.150***	-0.367***	-5.058***	-0.347***
	(0.065)	(0.023)	(0.408)	(0.021)	(0.057)	(0.017)	(0.415)	(0.016)
Zones (Base= North-Cent)								
North-East	0.126	0.043	0.040	0.003	0.365***	0.099***	2.409***	0.102**
	(0.118)	(0.041)	(0.490)	(0.042)	(0.127)	(0.032)	(0.661)	(0.045)
North-West	0.174	0.059	0.110	0.009	0.394***	0.106***	2.390***	0.102**
	(0.110)	(0.038)	(0.468)	(0.039)	(0.111)	(0.028)	(0.620)	(0.044)
South-East	0.166	0.057	0.402	0.028	-0.062	-0.020	-0.199	-0.021
	(0.134)	(0.045)	(0.548)	(0.038)	(0.091)	(0.029)	(0.476)	(0.050)
South-South	0.379***	0.121***	0.895**	0.049	-0.015	-0.005	-0.027	-0.003
	(0.110)	(0.036)	(0.451)	(0.031)	(0.091)	(0.029)	(0.470)	(0.047)
South-West	-0.008	-0.003	-0.534	-0.056	-0.516***	-0.186***	-1.790***	-0.201***
	(0.109)	(0.039)	(0.452)	(0.047)	(0.089)	(0.031)	(0.445)	(0.041)
Rural	-0.038	-0.012	-0.169	-0.013	-0.045	-0.015	0.142	0.015
	(0.075)	(0.024)	(0.305)	(0.022)	(0.061)	(0.020)	(0.285)	(0.031)

HH-size	0.014*	0.005*	0.034	0.003	0.016*	0.005*	0.059	0.006
	(0.009)	(0.003)	(0.036)	(0.003)	(0.009)	(0.003)	(0.037)	(0.004)
Own_home	0.058	0.019	0.060	0.005	-0.062	-0.020	-0.292	-0.031
	(0.073)	(0.024)	(0.273)	(0.022)	(0.060)	(0.020)	(0.233)	(0.024)
Electric	-0.149**	-0.048**	-0.625**	-0.044**	-0.096	-0.032	-0.261	-0.027
	(0.070)	(0.023)	(0.268)	(0.019)	(0.063)	(0.020)	(0.227)	(0.023)
Transport	-0.010	-0.003	-0.110	-0.009	0.020	0.007	0.049	0.005
	(0.084)	(0.028)	(0.258)	(0.021)	(0.071)	(0.024)	(0.224)	(0.024)
Micro-finance	0.061	0.020	-0.356	-0.031	-0.042	-0.014	0.280	0.028
	(0.102)	(0.033)	(0.300)	(0.030)	(0.091)	(0.031)	(0.278)	(0.026)
Profit	-0.000	-0.000	-0.000	-0.000	0.000	0.000	0.000	0.000
	(0.000)	(0.000)	(0.000)	(0.000)	(0.000)	(0.000)	(0.000)	(0.000)
Paid_hh	0.073	0.024	0.320	0.023*	0.053	0.018	0.021	0.002
	(0.066)	(0.021)	(0.199)	(0.014)	(0.056)	(0.018)	(0.164)	(0.018)
Unpaid_hh	0.168***	0.054***	0.334*	0.024*	0.089	0.029	0.288*	0.030*
	(0.063)	(0.020)	(0.194)	(0.014)	(0.057)	(0.019)	(0.166)	(0.017)
Constant	0.101		2.699***		0.789***		2.972***	
	(0.176)		(0.703)		(0.178)		(0.707)	
Log Likelihood	-1257		-1085.33		-1632.0		-1360	
$\hat{\rho}$ (rho)			0.960				0.952	
			(0.004)				(0.100)	
Likelihood ratio			343.39				545.0	
P-value			0.000				0.000	
Observations	2,278	2,278	2,278	2,278	3,116	3,116	3,116	3,116
Number of id			1,691				2,112	

Robust standard errors in parentheses; ***, ** and * significant at 1%, 5% and 10% respectively.

Note: AME=Average Marginal Effects.

Table A3: Determinants of Productivity Estimates for Informal HBEs using (Pooled OLS, Between Effects and Random Effects Models)

	Pooled OLS MODEL			RANDOM EFFECT MODEL		
	Wholesale and Retail Trade	Consultancy & Personal Services	Both	Wholesale and Retail Trade	Consultancy & Personal Services	Both
Variables	1	2	3	7	8	9
WRT (C&P =0)			-0.358***			-0.349***
			(0.065)			(0.069)
Age Group (Base <=25)						
26 – 35	0.072	0.579***	0.094	0.119	0.208	0.128
	(0.120)	(0.189)	(0.106)	(0.116)	(0.167)	(0.095)
36 – 45	0.133	1.222***	0.263**	0.188	0.442**	0.253***
	(0.118)	(0.251)	(0.102)	(0.120)	(0.181)	(0.098)
46 – 55	0.162	0.209	0.070	0.309**	0.103	0.242**
	(0.144)	(0.214)	(0.163)	(0.135)	(0.203)	(0.109)
> 55	-0.069	1.466***	0.051	0.127	0.243	0.160
	(0.160)	(0.359)	(0.150)	(0.134)	(0.223)	(0.107)
Education (Base = Others)						
Primary	0.271***	0.320**	0.285***	0.032	0.041	0.013
	(0.081)	(0.126)	(0.070)	(0.115)	(0.174)	(0.092)
Secondary	0.472***	0.850***	0.529***	0.332***	0.433**	0.326***
	(0.136)	(0.213)	(0.138)	(0.125)	(0.190)	(0.101)
Post-Secondary	0.007	-0.456**	-0.056	0.603***	1.006***	0.665***
	(0.110)	(0.192)	(0.089)	(0.160)	(0.252)	(0.137)
Male_owner	1.065***	-0.562*	0.895***	0.734***	0.587***	0.691***
	(0.181)	(0.305)	(0.173)	(0.089)	(0.130)	(0.074)
Zones (Base= North-Cent)						
North-East	-0.738***	0.136	-0.520**	-0.898***	-0.882***	-0.881***
	(0.168)	(0.328)	(0.219)	(0.147)	(0.255)	(0.134)
North-West	-0.231	0.564	-0.072	-0.483***	-0.730***	-0.566***
	(0.165)	(0.352)	(0.227)	(0.139)	(0.224)	(0.118)
South-East	-0.035	0.161	-0.074	0.024	-0.104	-0.015
	(0.159)	(0.305)	(0.154)	(0.166)	(0.298)	(0.144)
South-South	0.064	-0.684**	-0.197	0.419***	0.129	0.353***
	(0.205)	(0.291)	(0.311)	(0.132)	(0.231)	(0.114)
South-West	-0.409**	-2.431***	-0.790**	-0.114	-0.313	-0.168
	(0.196)	(0.578)	(0.330)	(0.140)	(0.216)	(0.114)
Rural	-0.098	-1.005***	-0.241*	0.035	-0.078	0.003
	(0.105)	(0.276)	(0.137)	(0.090)	(0.155)	(0.077)
HH-size	-0.043***	0.006	-0.040***	-0.034***	-0.022	-0.031***
	(0.011)	(0.021)	(0.009)	(0.010)	(0.018)	(0.010)
Electric	0.238***	-0.327*	0.151**	0.229***	-0.033	0.165**
	(0.079)	(0.174)	(0.069)	(0.082)	(0.141)	(0.073)
Transport	0.035	0.245*	0.057	0.044	0.163	0.064
	(0.089)	(0.138)	(0.076)	(0.094)	(0.150)	(0.075)

Micro-finance	0.148	0.925***	0.301**	0.078	0.578***	0.188*
	(0.123)	(0.255)	(0.137)	(0.109)	(0.182)	(0.110)
Turnover	0.050***	0.098***	0.060***	0.052***	0.113***	0.061***
	(0.013)	(0.035)	(0.013)	(0.011)	(0.027)	(0.013)
Paid_hh	-0.504***	-0.427***	-0.511***	-0.357***	-0.455***	-0.377***
	(0.080)	(0.120)	(0.065)	(0.070)	(0.116)	(0.061)
Inverse mills ratio (lambda)	**S**	**S**	**S**	**NS**	**NS**	**NS**
Constant	9.640***	14.583***	10.279***	8.532***	8.308***	8.598***
	(0.504)	(1.564)	(0.852)	(0.213)	(0.324)	(0.171)
Observations	1,547	526	2,073	1,547	526	2,073
R-squared	0.258	0.375	0.259	0.254	0.290	0.260
Number of id				1,160	427	1,553
LM Test Statistic				46.810	16.92	62.31
P-values				0.000	0.000	0.000

Robust standard errors in parentheses; ***, ** and * significant at 1%, 5% and 10% respectively. ; C&P=Consultancy and Personal Services; S implies that the selectivity term is significant in applied model, otherwise, NS (insignificant).

Table A4: Determinants of Productivity Estimates for Informal non-HBEs using (Pooled OLS and Random Effects Models)

	Pooled OLS MODEL			RANDOM EFFECT MODEL		
	Wholesale and Retail Trade	Consultancy & Personal Services	Both	Wholesale and Retail Trade	Consultancy & Personal Services	Both
Variables	1	2	3	7	8	9
C&P (WRT=0)			-0.432***			-0.394***
			(0.059)			(0.064)
Age Group (Base <=25)						
26 – 35	0.227**	0.660***	0.146*	0.209*	0.027	0.143*
	(0.107)	(0.169)	(0.087)	(0.120)	(0.153)	(0.086)
36 – 45	0.449***	1.286***	0.323***	0.406***	0.058	0.298***
	(0.105)	(0.227)	(0.087)	(0.120)	(0.160)	(0.089)
46 – 55	0.444***	0.228	0.375***	0.423***	0.235	0.344***
	(0.118)	(0.173)	(0.098)	(0.129)	(0.175)	(0.102)
> 55	0.451***	1.070***	0.308***	0.479***	0.034	0.342***
	(0.122)	(0.226)	(0.102)	(0.134)	(0.185)	(0.105)
Education (Base = Others)						
Primary	-0.043	-0.913***	0.029	-0.001	0.214	0.050
	(0.132)	(0.182)	(0.113)	(0.133)	(0.225)	(0.120)
Secondary	0.237*	-1.465***	0.254**	0.230*	0.288	0.237*
	(0.139)	(0.328)	(0.119)	(0.137)	(0.225)	(0.124)
Post-Secondary	0.643***	-3.862***	0.629***	0.642***	0.566**	0.616***
	(0.157)	(0.577)	(0.135)	(0.159)	(0.266)	(0.142)
Male_owner	0.467***	1.341***	0.504***	0.453***	0.559***	0.488***
	(0.071)	(0.153)	(0.058)	(0.077)	(0.114)	(0.064)
Zones (Base= North-Cent)						
North-East	-0.471***	3.640***	-0.411***	-0.464***	-0.218	-0.423***
	(0.161)	(0.609)	(0.133)	(0.157)	(0.249)	(0.141)
North-West	-0.089	1.052***	-0.168	-0.051	-0.360	-0.141
	(0.146)	(0.312)	(0.121)	(0.143)	(0.223)	(0.128)
South-East	-0.400***	-1.797***	-0.298***	-0.403***	0.018	-0.291***
	(0.102)	(0.321)	(0.085)	(0.112)	(0.177)	(0.090)
South-South	0.069	-0.828***	0.146*	0.085	0.376**	0.164*
	(0.104)	(0.241)	(0.087)	(0.112)	(0.177)	(0.092)
South-West	-0.260**	-5.174***	-0.245***	-0.245**	-0.201	-0.232**
	(0.109)	(0.766)	(0.088)	(0.113)	(0.164)	(0.094)
Rural	-0.161**	-2.014***	-0.135**	-0.160**	-0.172	-0.144**
	(0.069)	(0.312)	(0.060)	(0.075)	(0.123)	(0.066)
HH-size	0.000	-0.090***	-0.000	0.001	-0.010	-0.002
	(0.010)	(0.020)	(0.008)	(0.011)	(0.017)	(0.009)
Electric	0.192***	0.342***	0.189***	0.171**	0.084	0.155**
	(0.072)	(0.114)	(0.061)	(0.072)	(0.112)	(0.061)
Transport	0.060	0.056	0.061	0.084	0.081	0.081
	(0.078)	(0.108)	(0.064)	(0.077)	(0.113)	(0.061)

Micro-finance	-0.063	0.340**	-0.010	-0.140	0.121	-0.064
	(0.110)	(0.133)	(0.087)	(0.103)	(0.139)	(0.085)
Turnover	0.004***	0.004	0.004***	0.004***	0.004	0.004***
	(0.000)	(0.003)	(0.000)	(0.001)	(0.005)	(0.000)
Paid_hh	-0.457***	-0.246**	-0.484***	-0.318***	-0.410***	-0.352***
	(0.065)	(0.105)	(0.053)	(0.060)	(0.086)	(0.050)
Inverse mills ratio (lambda)	**NS**	**S**	**NS**	**NS**	**NS**	**NS**
Constant	9.023***	20.371***	9.027***	8.984***	8.681***	9.024***
	(0.199)	(1.759)	(0.170)	(0.210)	(0.346)	(0.179)
Observations	2,012	778	2,790	2,012	778	2,790
R-squared	0.134	0.198	0.136	0.132	0.136	0.133
Number of id				1,406	585	1,937
LM-Test statistic				71.89	21.08	104.24
P-values				0.000	0.000	0.000

Robust standard errors in parentheses; ***, ** and * significant at 1%, 5% and 10% respectively; WRT=Wholesale, Retail and Trade; C&P=Consultancy and Personal Services; S implies that the selectivity term is significant in applied model, otherwise, NS (insignificant).

Table A5: Coefficients of the selectivity terms from all models

	**Pooled OLS**			**RANDOM EFFECTS MODEL**		
	Wholesale and Retail Trade	Consultancy & Personal Services	Both	Wholesale and Retail Trade	Consultancy & Personal Services	Both
**HBE and non-HBE (Combined)**						
Inverse mills ratio (lambda)	-0.072	-3.007	-0.952	1.250***	0.167	0.894**
	(0.565)	(2.064)	(0.883)	(0.462)	(0.666)	(0.382)
**HBE**						
Inverse mills ratio(lambda)	-1.461**	-6.180***	-3.091**	0.236	-0.184	-0.267
	(0.632)	(1.547)	(1.478)	(0.217)	(0.191)	(0.581)
**Non-HBE**						
Inverse mills ratio(lambda)	-0.057	-13.164***	0.854	0.458	0.009	2.878
	(0.749)	(1.948)	(1.046)	(0.324)	(0.115)	(2.134)

Robust standard errors in parentheses; ***, ** and * significant at 1%, 5% and 10% respectively
