# Supplementary material for: Participation and returns from informal service-oriented non-farm enterprises: Evidence from a survey of Nigerian households
Source: PLoS One. 2024 Mar 18;19(3):e0298794. doi: 10.1371/journal.pone.0298794 (PMC10947675; doi:10.1371/journal.pone.0298794)
Supplement: Attachment — Submitted filename: Comment to reviewers.docx [file pone.0298794.s004.docx]

We are grateful for the constructive feedback, which greatly contributed to the enhancement of the manuscript and have addressed each point meticulously in the revised manuscript.

1. ***Policy Recommendations in Abstract***: We have incorporated policy recommendations into the abstract as follows:

***“***Key recommendations stemming from this study include addressing gender-based segregation and economic disparities, prioritising financial inclusion for small business development, bridging infrastructure gaps, and implementing policies that acknowledge and bolster the informal sector.”

2. ***Addressed Comments on clarity and copyediting***: We have thoroughly addressed all the comments raised by the reviewers, ensuring clarity, accuracy, and completeness throughout the manuscript. Each reviewer's concern has been carefully considered and incorporated as appropriate.
3. ***Separate Captions for Figures***: Captions for each figure have been added as a separate section in the manuscript to comply with the journal's requirements.
4. ***Tables in Main Manuscript***: All tables have been integrated into the main manuscript as per the instructions. Supplementary tables have been uploaded separately as "supporting information" files.
5. ***Updated Reference List***: We have reviewed and revised the reference list to ensure accuracy and completeness, adhering strictly to the PLOS ONE format. Retracted papers have been addressed in accordance with the journal's guidelines.
